# Mechanically Robust Poly(ionic liquid) Block Copolymers as Self-Assembling Gating Materials for Single-Walled Carbon-Nanotube-Based Thin-Film Transistors

**DOI:** 10.1021/acsapm.2c02223

**Published:** 2023-03-30

**Authors:** Daniil R. Nosov, Bahar Ronnasi, Elena I. Lozinskaya, Denis O. Ponkratov, Laura Puchot, Patrick Grysan, Daniel F. Schmidt, Benoît H. Lessard, Alexander S. Shaplov

**Affiliations:** † Luxembourg Institute of Science and Technology (LIST), 5 Avenue des Hauts-Fourneaux, L-4362 Esch-sur-Alzette, Luxembourg; ‡ Department of Physics and Materials Science, University of Luxembourg, 2 Avenue de l’Université, L-4365 Esch-sur-Alzette, Luxembourg; § Department of Chemical & Biological Engineering, University of Ottawa, 161 Louis Pasteur, Ottawa, Ontario K1N 6N5, Canada; ∥ A.N. Nesmeyanov Institute of Organoelement Compounds Russian Academy of Sciences (INEOS RAS), Vavilov str. 28, bld. 1, 119334 Moscow, Russia; ⊥ School of Electrical Engineering and Computer Science, University of Ottawa, 800 King Edward Avenue, Ottawa, Ontario K1N 6N5, Canada

**Keywords:** poly(ionic liquid), polyelectrolyte, ionic conductivity, block copolymer self-assembly, capacitor, thin-film transistors

## Abstract

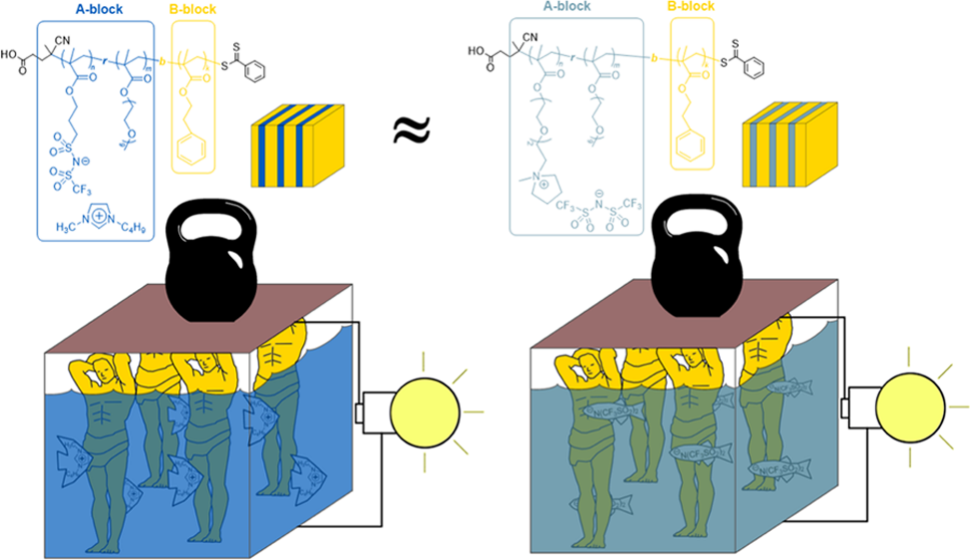

The proliferation of high-performance thin-film electronics depends on the development of highly conductive solid-state polymeric materials. We report on the synthesis and properties investigation of well-defined cationic and anionic poly(ionic liquid) AB–C type block copolymers, where the AB block was formed by random copolymerization of highly conductive anionic or cationic monomers with poly(ethylene glycol) methyl ether methacrylate, while the C block was obtained by post-polymerization of 2-phenylethyl methacrylate. The resulting ionic block copolymers were found to self-assemble into a lamellar morphology, exhibiting high ionic conductivity (up to 3.6 × 10^–6^ S cm^–1^ at 25 °C) and sufficient electrochemical stability (up to 3.4 V vs Ag^+^/Ag at 25 °C) as well as enhanced viscoelastic (mechanical) performance (storage modulus up to 3.8 × 10^5^ Pa). The polymers were then tested as separators in two all-solid-state electrochemical devices: parallel plate metal–insulator–metal (MIM) capacitors and thin-film transistors (TFTs). The laboratory-scale truly solid-state MIM capacitors showed the start of electrical double-layer (EDL) formation at ∼10^3^ Hz and high areal capacitance (up to 17.2 μF cm^–2^). For solid-state TFTs, low hysteresis was observed at 10 Hz due to the completion of EDL formation and the devices were found to have low threshold voltages of −0.3 and 1.1 V for p-type and n-type operations, respectively.

## 1 Introduction

Over the last two decades, poly(ionic liquid)s (PILs) or polymeric ionic liquids have become very promising materials for use in next-generation batteries, fuel cells, and printed electronics.^1−4^ Electrochemical properties of PILs can be improved through structural modifications such as the introduction of the ionic species with high charge delocalization,^5^ asymmetric anions,^6^ and flexible spacers between the backbone and the bonded ion that contain oxyethylene chains^7,8^ or other polar groups.^2,9^ PILs can also exhibit high electrochemical stability (up to 5.5 V vs Li+/Li), thermal stability (up to 350 °C), ionic conductivity (up to 10^–4^ S cm^–1^ at 25 °C, see Table S1), and excellent processability.^1,4,9−12^ In most cases, the ionic conductivity decreases with increase in glass transition temperature (*T*_g_), thus making it challenging to obtain both high electrochemical performance and good mechanical stability.^1,13^ One approach to overcome this problem was introduced by Elabd, Winey, and others^12,14−18^ through the use of block copolymer self-assembly where a soft ion transport domain is paired with a mechanically stable domain.^12,14−16^ For example, Ye et al. demonstrated that block copolymers of 1-[(2-methacryloyloxy)ethyl]-3-butylimidazolium bis(trifluoromethanesulfonyl) imide (MEBIm-TFSI) and methyl methacrylate exhibited microphase separation and outperformed random copolymers made from the same monomers by two orders of magnitude difference in ionic conductivity.^17^ Similar acrylate-based ionic block copolymers demonstrated ∼1.5–2 orders of magnitude higher ionic conductivity when a strong microphase separation was demonstrated compared with weak microphase separation.^18^ Moreover, the improvement in conductivity was found to be dependent on the nature of the microphase separation and its orientation with respect to the ion transport direction.^14^

Contrary to cationic PIL block copolymers, anionic PIL block copolymers are not as well studied in the literature, with the majority containing Li^+^ as a counterion.^19−24^ For example, Balsara et al.^21^ reported A–B type poly(ethylene oxide)-*b*-poly(styrenesulfonyllithium(trifluoromethylsulfonyl)imide) (PEO-*b*-PSLiTFSI) copolymers with lamellar phase separation and conductivities as high as 10^–4^ when heating above 50 °C. Balsara,^23^ Bouchet,^19^ and Porcarelli^25^ reported the synthesis of B–A–B triblock copolymers composed of one PEO block and two lithium 1-[3-(methacryloyloxy)propylsulfonyl]-1-(trifluoromethylsulfonyl)imide (MLiTFSI)-based blocks. These triblock copolymers demonstrated high conductivity only above the melting point of the PEO block (*T* > 55 °C), leading to a breakdown of the observed lamellar morphology. Thus, A–B and B–A–B block copolymers did not benefit from the microphase separation that can be explained by (a) low conductivity in neat PSLiTFSI and PMLiTFSI blocks due to high *T*_g_ and the absence of the polar solvating groups around the bonded anion and (b) the solid nature of the PEO block at RT that cannot improve the dissociation of cations. To maintain the desired block copolymer self-assembly, AB–C block copolymers were designed with an AB block represented by a random copolymer of anionic ILMs with poly(ethylene glycol) methyl ether methacrylate (PEGM), a low *T*_g_ monomer bearing short side PEG chains and a C block as a neutral polymer with high *T*_g_ for improved mechanical performance. Following this strategy, we reported the preparation of anionic poly[TMC*_n_*-*b*-(MLiTFSI*_m_*-*r*-PEGM*_k_*)], where the mechanically robust block was obtained by ring-opening polymerization of trimethylene carbonate (TMC).^26^ These block copolymers evolved in quasi-hexagonally packed cylinder morphology showed ionic conductivity similar to the disordered random poly(MLiTFSI-*r*-PEGM) copolymers (2.9 × 10^–7^ S cm^–1^ at 25 °C) along with improved viscoelastic properties and an outstanding stability vs anodic oxidation (exceeding 4.8 V vs Li^+^/Li at 70 °C).^26^ Long et al. followed with the report of C–AB–C triblock copolymers characterized by an ionic conductivity of 10^–6^ S cm^–1^ at 25 °C that was attributed to the optimized ionic lamellar phase separation and phase percolation.^20^ Lastly, Lozinskaya et al.^27^ reported [(MLiTFSI*_n_-r-*PEGM)*_m_*]-*b*-(PhEtM)*_k_* copolymers with a second high *T*_g_ block based on 2-phenylethyl methacrylate (PhEtM), showing promising lamellar phase separation, good mechanical properties, and high ionic conductivity (4.1 × 10^–7^ S cm^–1^ at 25 °C).

Thin-film transistors (TFTs) represent electrical switches, which are essential in many applications, including sensors, light management in displays, and other basic circuitry. For TFT functioning using printed batteries, a low operating voltage is required. While the realization of such low operating voltages would normally demand very low layer thicknesses that are very challenging to print at high speeds, in the case of PILs, these materials form an electrical double layer (EDL), which leads to a thickness-independent, high capacitance material capable of working under low operating voltages without requiring ultrathin layers. Furthermore, enhancements in PIL conductivity promise increased switching speeds.^28^ Finally, we have recently demonstrated additional performance benefits of ionic block copolymer self-assembly as applied to the formation of gating material layers in TFTs.^29^

**Figure 1:**
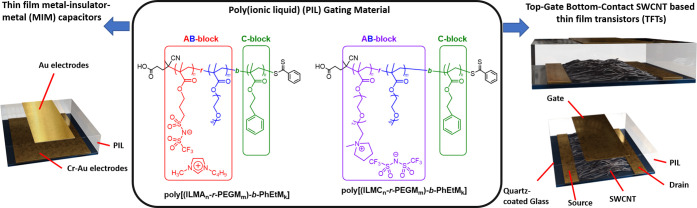
**Poly[(ILM*_n_-r-*PEGM*_m_*)-*b*-PhEtM*_k_*]** block copolymers used in thin-film metal–insulator–metal (MIM) capacitors and single-walled carbon-nanotube (SWCNT)-based thin-film transistors (TFTs).

In this work, we designed and synthesized three novel ionically conductive and mechanically robust block copolymers with AB–C type architecture (Figure 1). The AB blocks, responsible for ion conduction, were prepared by RAFT random copolymerization of highly conductive anionic (ILMA) or cationic (ILMC) monomers with PEGM. The C block, accountable for the mechanical properties of copolymers, was synthesized by post-polymerization of 2-phenylethyl methacrylate (PhEtM). The resultant block copolymers exhibited high ionic conductivity (up to 3.6 × 10^–6^ S cm^–1^ at 25 °C) and showed lamellar microphase separation that led to significant improvement in their dimensional stability and viscoelastic properties in comparison with the corresponding random AB copolymers. These cationic and anionic block copolymers were further integrated into all-solid-state thin-film double-plate metal–insulator–metal (MIM) capacitors and thin-film transistors (TFTs). In MIMs, ionic block copolymers were capable to display the formation of a prominent EDL at low frequencies (10^3^ Hz) and demonstrated as high maximum areal capacitance as 17.2 μF cm^–2^ (at 25 °C). The integration of block copolymers into the single-walled carbon-nanotube-based TFTs resulted in either n-type or p-type operation with low threshold voltages (<1.5 V) and small device hysteresis. This study provides insight into structure–property relationships for PILs, which will enable future adoption of low-operation flexible thin-film electronics.

## 2 Experimental Section

### 2.1 Materials

Poly(ethylene glycol)methyl ether methacrylate (PEGM, *M*_n_ = 500 g/mol, Aldrich), 1-butyl-3-methylimidazolium bromide (99%, Iolitec), magnesium sulfate (MgSO_4_, anhydrous, 99.5%, Aldrich), 4-methoxyphenol (99%, Aldrich), 2-phenylethyl methacrylate (PhEtM, 98%, Jinan Yudong Technology Co., Ltd.), 2-[2-(chloroethoxy)-ethoxy]ethanol (>98%, TCI Europe), lithium bis(trifluoromethylsulfonyl)imide (LiTFSI, >99%, Solvionic), *N*-methylimidazole (>99%, redistilled, Aldrich), 3-sulfopropyl methacrylate potassium salt (98%, Aldrich), 4-cyano-4-(phenylcarbonothioylthio)pentanoic acid (CPAD, chain-transfer agent (CTA), >97%, Aldrich), octadecyltrichlorosilane (OTS, ≥90%, Aldrich), dichloromethane (DCM, 99.8%, Aldrich), dimethylformamide (DMF, anhydrous, 99.5%, Acros), diethyl ether (99%, Aldrich), methanol (99.8%, Aldrich), golden leafs/foil (22 carat, Carl ROTH), chromium-coated tungsten rods (Cr, Angstrom Engineering), and gold (Au, 99.99%, Angstrom Engineering) were used as received. Single-walled carbon nanotubes (SWCNTs, Raymor Industries, diameter ∼ 1.5 nm, length 0.3–4.0 μm) were purified using a poly(9,9′-didodecylfluorene-*co*-*N*-(2′-decyltetradecane)-carbazole) polymer (PCPF) in toluene following previously reported procedures.^30,31^ Methacryloyl chloride (>97%, Acros) and thionyl chloride (99.7% Acros) were distilled over linseed oil. *N*-Methylpyrrolidine (97%, Aldrich) was distilled under vacuum prior to use. 2,2′-Azobisisobutyronitrile (AIBN, initiator, 98%, Acros) was recrystallized from methanol. 4-Methoxyphenol (99%, Acros) was sublimed in vacuum prior to use. Lithium 1-[3-(methacryloyloxy)propylsulfonyl]-1-(trifluoromethanesulfonyl)imide was synthesized in accordance with the procedures published previously.^32,33^

### 2.2 Ionic Liquid-Like Monomers (ILMs) Synthesis

#### 2.2.1 1-[2-(2-(2-(Methacryloyloxy)ethoxy)ethoxy)ethyl]-*N*-methylpyrrolidinium bis(trifluoromethylsulfonyl)imide (Cationic ILM or **ILMC**)

The synthesis of **ILMC** was performed via three reaction steps: (i) alkylation of *N*-methylpyrrolidinium by 2-[2-(chloroethoxy)-ethoxy]ethanol, (ii) acylation of *N*-[2-(2-(2-(hydroxy)ethoxy)ethoxy)ethyl]-*N*-methylpyrrolidinium chloride with methacryloyl chloride in the presence of a small excess of triethylamine, and (iii) ionic metathesis of the chloride ionic monomer with lithium bis(trifluoromethylsulfonyl)imide in full accordance with the procedure published previously.^34,35^ Calcd for C_17_H_28_F_6_N_2_O_8_S_2_ (566.53): C, 36.04%; H, 4.98%; N, 4.94%; found: C, 36.17%; H, 5.15%; N, 4.94%; *T*_g_ = −67 °C (DSC, 2 °C min^–1^); ^1^H NMR (400 MHz, CDCl_3_): δ = 6.04 (s, 1H, CH_2_=C *Z*), 5.54 (m, 1H, CH_2_=C *E*), 4.22 (m, 2H, COOCH_2_), 3.84 (br. m, 2H, OCH_2_CH_2_N), 3.66 (m, 2H, COOCH_2_CH_2_), 3.60 (m, 4H, OCH_2_CH_2_O), 3.52 (m, 6H, OCH_2_CH_2_N, CH_2_-2,5), 3.04 (s, 3H, N–CH_3_), 2.17 (br. m, 4H, CH_2_-3,4), 1.87 (s, 3H, CH_3_–C=) ppm; ^13^C NMR (100 MHz, CDCl_3_): δ = 167.2 (C=O), 135.9 (CH_2_=C(CH_3_)), 125.7 (CH_2_=C(CH_3_)), 123.9–114.8 (q, ^1^*J*_CF_ = 321 Hz), 70.3, 70.0, 68.9 (OCH_2_), 65.4 (2C, C-2,5), 64.8, 63.6, 63.2 (OCH_2_) 48.5 (CH_3_–N), 21.1 (2C, C-3,4), 18.0 (CH_2_=C(CH_3_)) ppm; ^19^F NMR (376.5 MHz, CDCl_3_): δ = −79.1 (s, CF_3_) ppm; IR (KBr pellet): 2957 (m, ν_C–H_), 2893 (m, ν_C–H_), 1717 (vs, ν_CO_), 1637 (m, ν_C=C_), 1475 (m), 1456 (m), 1354 (vs, ν_asSO_2__), 1331 (m), 1299 (m), 1227 (m), 1193 (vs, ν_CF_), 1138 (s, ν_sSO2_), 1058 (vs, ν_CF_), 1000 (w), 937 (w), 789 (m), 740 (m), 654 (m), 618 (s), 571 (s), 515 (m), 455 (w) cm^–1^; η = 258 cP (25 °C); ρ = 1.41 g cm^–3^ (25 °C); σ_DC_ = 5.5 × 10^–4^ S cm^–1^ (25 °C).

#### 2.2.2 1-Butyl-3-methylimidazolium 1-[3-(Methacryloyloxy) propylsulfonyl]-1-(trifluoromethanesulfonyl)imide (Anionic ILM or **ILMA**)

**ILMA** was synthesized from lithium 1-[3-(methacryloyloxy)propylsulfonyl]-1-(trifluoromethanesulfonyl)imide via an ion metathesis reaction with an excess of 1-butyl-3-methylimidazolium bromide in an aqueous medium.

The solution of lithium 1-[3-(methacryloyloxy)propylsulfonyl]-1-(trifluoromethanesulfonyl) imide (32.50 g, 74.00 mmol) in 150 ml of milli-Q water was added dropwise to the aqueous solution (60 mL) of 1-butyl-3-methylimidazolium bromide (21.10 g, 96.20 mmol) at room temperature. The formation of an emulsion was observed immediately, and the stirring was continued for 2 h at RT. The organic oil was extracted from the reaction mixture with dichloromethane. The DCM layer was washed with water (4 × 30 mL) and dried over anhydrous magnesium sulfate. MgSO_4_ was filtered off, a catalytic amount of 4-methoxyphenol (as an inhibitor) was added and dichloromethane was removed under reduced pressure at RT. The resultant light-yellow transparent oil was dried at 25 °C/0.1 mbar for 8 h. Yield: 27.40 g (77.4%). Calcd for C_16_H_26_F_3_N_3_O_6_S_2_ (477.51): C, 40.25%; H, 5.49%; N, 8.80%; found: C, 40.11%; H, 5.40%; N, 8.92%; *T*_g_ = −63 °C (DSC, 2 °C min^–1^); ^1^H NMR (600.2 MHz, DMSO-*d*_6_): δ = 9.09 (s, 1H, −N=CH–N), 7.76 (s, 1H, −(CH_3_)N–CH=CH–N(CH_2_)), 7.69 (s, 1H, −(CH_3_)N–CH=CH–N(CH_2_)), 6.03 (s, 1H, CH_2_=C(CH_3_)), 5.67 (s, 1H, CH_2_=C(CH_3_)), 4.17 (dt, *J = 13.4, 6.8 Hz*, 4H, −CH_2_–N, −CO–O–CH_2_), 3.85 (s, 3H, −N(CH_3_)), 3.04–3.10 (m, 2H, −CH_2_–SO_2_), 1.97–2.06 (m, 2H, −CH_2_–CH_2_–SO_2_), 1.88 (s, 3H, CH_2_=C(CH_3_)), 1.74–1.79 (m, 2H, −CH_2_–CH_2_–CH_3_), 1.23–1.29 (m, 2H, −CH_2_–CH_2_–CH_3_), 0.90 (t, *J* = 7.4 Hz, 3H, −CH_2_–CH_2_–CH_3_) ppm; ^13^C NMR (150.9 MHz, DMSO-*d*_6_): δ = 166.4, 136.5, 135.8, 125.6, 123.6, 122.2, 120.1 (q, *J*_CF_ = 324.5 Hz), 62.8, 51.3, 48.5, 35.7, 31.3, 23.5, 18.7, 17.9, 13.1 ppm; ^19^F NMR (564.7 MHz, DMSO-*d*_6_): δ = −79.80 (s, CF_3_) ppm; FTIR (ATR mode): 3151 (m), 3114 (m), 2963 (m, ν_CH_), 2938 (m, ν_CH_), 2877 (w, ν_CH_), 1716 (s, ν_C=O_), 1637 (m, ν_C=C_), 1572 (m), 1466 (m, ν_CH_), 1410 (w), 1321(vs, ν_asSO_2__), 1298 (s), 1222 (m, ν_CF_), 1178 (vs, ν_symSO_2__), 1123 (s), 1052 (s, ν_CF_), 1023 (m), 945 (9w), 817 (m), 754 (m), 712 (w), 649 (m), 622 (s) cm^–1^; η = 504 cP (25 °C); ρ = 1.31 g cm^–3^ (25 °C); σ_DC_ = 2.4 × 10^–4^ S cm^–1^ (25 °C).

### 2.3 Synthesis of Random **Poly(ILM*_n_-r-*PEGM*_m_*)** Macro-Chain-Transfer Agents

Random copolymers **poly(ILMA***_**n**_**-r-*****PEGM***_**m**_***)** and **poly(ILMC***_**n**_**-r-*****PEGM***_**m**_***)** were prepared via RAFT copolymerization and were further used as macro-chain-transfer agents (macro-CTAs) for further synthesis of block copolymers. While detailed loadings for the syntheses of **poly(ILMA**_**12**_***-r-*****PEGM**_**68**_**)** and **poly(ILMC**_**24**_***-r-*****PEGM**_**40**_**)** are presented in Table S2, the typical polymerization procedure is given below by the example of **poly(ILMC**_**12**_***-r-*****PEGM**_**68**_**)** synthesis.

#### 2.3.1 Poly(ILMC_12_*-r-*PEGM_68_)

PEGM (1.02 g, 2.04 mmol) and **ILMC** (0.23 g, 0.41 mmol) were dissolved in 3.2 mL (3.00 g) of anhydrous DMF at RT, whereupon AIBN (0.87 mg, 5.30 μmol) and CPAD (7.40 mg, 26.50 μmol) were added, and the stirring was continued until the formation of a clear solution. From this loading, the following initial reagent ratios can be calculated: ([PEGM]_o_ + [ILMC]_o_)/[CPAD]_o_/[AIBN]_o_ = 92.4:1:0.2. The solution was quantitatively transferred to a Schlenk flask equipped with a magnetic stirring bar, degassed by three freeze–evacuate–thaw cycles, flashed with argon, and placed in a bath preheated to 60 °C. Polymerization was continued at 60 °C for 15 h. The resultant viscous solution was cooled to RT, diluted with DMF, and precipitated into excess of diethyl ether. The copolymer was collected, redissolved in milli-Q water, and dialyzed against water for 2 days. 4-Methoxyphenol (polymerization inhibitor, 40 mg) was added to the obtained aqueous solution and the copolymer was isolated by freeze-drying. The final drying was carried out at 50 °C/0.1 mbar for 12 h in a B-585 oven (Buchi Glass Drying Oven, Switzerland) filled with P_2_O_5_. The product in the form of a pink viscous cold flowing liquid was stored at 5 °C before further use. Yield: 0.90 g (72.0%); the total conversion determined by ^1^H NMR: *q* = 0.86; ^1^H NMR (400.1 MHz, CDCl_3_): δ = 4.08–4.10 (t, 13.6H, −CO–O–CH_2_ PEGM and ILMC), 3.56–3.65 (m, 211.2H, −CH_2_–O–CH_2_–CH_2_–O-PEGM and ILMC, OCH_2_CH_2_N ILMC, OCH_2_CH_2_N ILMC, CH_2_-2,5 ILMC), 3.38 (s, 17.4H, −O–CH_3_ PEGM), 3.17 (s, 3H, −N–CH_3_), 2.19–2.39 (m, 4H, CH_2_-3,4 ILMC), 0.72–2.15 ((m, 34.0H, −CH_2_–C(CH_3_)) PEGM and ILMC, −CH_2_–C(CH_3_) PEGM and ILMC) (the ratio between **ILMC** and PEGM in the copolymer was determined by ^1^H NMR as 1:5.7 by mol) ppm; ^19^F NMR (376.5 MHz, CDCl_3_): δ = −78.75 (s, CF_3_) ppm; FTIR (ATR mode): 2873 (vs, ν_C–H_), 1729 (vs, ν_C=O_), 1455 (m), 1353 (s, ν_asSO_2__), 1248 (m), 1193 (vs, ν_CF_), 1135 (vs, ν_sSO2_), 1114 (vs, ν_C–O–C_), 1107 (vs), 951 (m), 854 (m), 618 (m), 570 (m), 514 (m) cm^–1^; *M*_n(SEC)_ = 29.6 kDa, *M*_w_/*M*_n_ = 1.11; *M*_n(NMR)_ = 40.8 kDa; *T*_g_ = −54 °C (DSC 5 °C min^–1^); *T*_onset_ = 155 °C (TGA, 5 °C min^–1^, on air); σ_DC_ = 9.3 × 10^–6^ S cm^–1^ (25 °C).

**Poly(ILMC**_**24**_***-r-*****PEGM**_**40**_**)** was obtained as a pink soft and sticky mass. Yield: 0.78 g (72.0%). Total conversion determined by ^1^H NMR: *q* = 0.86; ^1^H NMR (400.1 MHz, CDCl_3_): δ = 4.08–4.10 (t, 5.4H, −CO–O–CH_2_ PEGM and ILMC), 3.56–3.65 (m, 71.1H, −CH_2_–O–CH_2_–CH_2_–O-PEGM and ILMC, OCH_2_CH_2_N ILMC, OCH_2_CH_2_N ILMC, CH_2_-2,5 ILMC), 3.38 (s, 5H, −O–CH_3_ PEGM), 3.17 (s, 3H, −N–CH_3_), 2.19–2.39 (m, 4H, CH_2_-3,4 ILMC), 0.72–2.15 ((m, 13.4H, −CH_2_–C(CH_3_)) PEGM and ILMC, −CH_2_–C(CH_3_) PEGM and ILMC) (the ratio between **ILMC** and PEGM in the copolymer was determined by ^1^H NMR as 1:1.7 by mol) ppm; ^19^F NMR (376.5 MHz, CDCl_3_): δ = −78.79 (s, CF_3_) ppm; FTIR (ATR mode): 2876 (s, ν_C–H_), 1729 (s, ν_C=O_), 1456 (m), 1354 (vs, ν_asSO_2__), 1334 (s), 1227 (m), 1192 (vs, ν_CF_), 1135 (vs, ν_sSO2_), 1114 (vs, ν_C–O–C_), 1059 (vs, ν_CF_), 950 (m), 854 (m), 788 (m), 618 (m), 571 (m), 514 (m) cm^–1^; *M*_n(SEC)_ = 21.3 kDa, *M*_w_/*M*_n_ = 1.10; *M*_n(NMR)_ = 33.6 kDa; *T*_g_ = −37 °C (DSC 5 °C min^–1^); *T*_onset_ = 160 °C (TGA, 5 °C min^–1^, on air); σ_DC_ = 1.6 × 10^–5^ S cm^–1^ (25 °C).

**Poly(ILMA**_**12**_***-r-*****PEGM**_**68**_**)** was isolated as a pink sticky mass. Yield: 8.10 g (87.0%); Total conversion determined by ^1^H NMR: *q* = 0.94; ^1^H NMR (600.2 MHz, DMSO-*d*_6_): δ = 9.07 (s, 1H), 7.74 (s, 1H), 7.68 (s, 1H), 4.16 (t, *J* = 7.2 Hz, 2H), 4.01 (br. s, 4H), 3.85 (s, 3H), 3.80–3.42 (m, 39H), 3.24 (s, 3H), 3.00 (s, 2H), 2.19–1.31 (m, 6H), 1.77 (p, *J* = 7.4 Hz, 2H), 1.27 (sext, *J* = 7.4 Hz, 2H), 1.25–0.45 (m, 6H), 0.90 (t, *J* = 7.4 Hz, 3H) (the ratio between **ILMA** and PEGM in the copolymer was determined by ^1^H NMR as 1:5.8 by mol) ppm; ^13^C NMR (150.9 MHz, DMSO-*d*_6_): δ = 176.7, 136.5, 123.6, 122.2 120.1 (q, *J*_CF_ = 324.3 Hz), 71.3, 69.8, 69.56, 67.8, 63.8, 58.0, 51.1, 48.5, 35.7, 31.3, 23.01, 18.7, 16.6, 13.2 ppm; ^19^F NMR (564.7 MHz, DMSO-*d*_6_): δ = −79.8 (s) ppm; IR (ATR mode): 3151 (w), 3111 (w), 2871 (s, ν_CH_), 1727 (s, ν_C=O_), 1572 (w), 1454 (m, ν_CH_), 1387 (w), 1351 (w), 1322 (s, ν_asSO_2__), 1298 (m), 1248 (m, ν_as–C–O–C–_), 1224 (m, ν_CF_), 1177 (vs, ν_sSO2_), 1120 (vs, ν_–C–O–_), 1054 (s, ν_CF_), 949 (m), 852 (m), 621 (s) cm^–1^; *M*_n(SEC)_ = 39.4 kDa, *M*_w_/*M*_n_ = 1.35; *M*_n(NMR)_ = 39.8 kDa; *T*_g_ = −40 °C (DSC 5 °C min^–1^); *T*_onset_ = 165 °C (TGA, 5 °C min^–1^, on air); σ_DC_ = 7.6 × 10^–6^ S cm^–1^ (25 °C).

### 2.4 Synthesis of **Poly[(ILM_*n*_*-r-*PEGM*_m_*)-*b*-PhEtM*_k_*]** Block Copolymers

**Poly[(ILM**_***n***_*-**r**-***PEGM***_**m**_***)-*****b*****-PhEtM***_**k**_***]** block copolymers were synthesized via RAFT polymerization of 2-phenylethyl methacrylate in the presence of the respective **poly(ILM***_**n**_**-r-*****PEGM***_**m**_***)** macro-CTA. The typical polymerization procedure is given below by the example of **poly[(ILMC**_**12**_***-r-*****PEGM**_**68**_**)-*****b*****-(PhEtM)**_**97**_**]** synthesis, while the detailed loadings for the preparation of **poly[(ILMA**_**12**_***-r-*****PEGM**_**68**_**)-*****b*****-(PhEtM)**_**96**_**]** and **poly[(ILMC**_**24**_***-r-*****PEGM**_**40**_**)-*****b*****-(PhEtM)**_**95**_**]** are presented in Table S3.

#### 2.4.1 Poly[(ILMC_12_*-r-*PEGM_68_)-*b*-(PhEtM)_97_]

The solution of **poly(ILMC_12_*-r-*PEGM_68_)** macro-CTA (0.50 g, 12.25 μmol), PhEtM (0.46 g, 2.39 mmol), and AIBN (0.40 mg, 2.45 μmol) in 3.00 mL of anhydrous DMF (2.87 g) with an initial reagents ratio of [PhEtM]_o_/[macro-CTA]_o_/[AIBN]_o_ = 195:1:0.2 was quantitatively transferred to the Schlenk flask equipped with a magnetic stirring bar, degassed by three freeze–evacuate–thaw cycles, flashed with argon, and placed in the bath preheated to 60 °C. Polymerization proceeded at 60 °C for 15 h, whereupon the reaction was stopped by rapid cooling with liquid nitrogen. The obtained viscous polymer solution was diluted with dichloromethane and precipitated twice in a large excess of diethyl ether. The obtained block copolymer was dried at 25 °C/0.1 mbar for 24 h and then at 50 °C/0.1 mbar for 24 h in B-585 oven filled with P_2_O_5_ to give a slightly pink solid wax material. Yield: 0.56 g (59.0%); calcd for (C_2945_H_4699_F_72_N_25_O_1040_S_26_): C, 59.65%; H, 7.99%; found: C, 58.95%; H, 8.11%; conversion of the PhEtM determined by ^1^H NMR: *q* = 0.50; ^1^H NMR (400.1 MHz, CDCl_3_): δ = 7.24–7.33 (m, 41.7H, CH_2_–Ph PhEtM), 4.14–4.21 (m, 30.3H, −CO–O–CH_2_ ILMC, PEGM and PhEtM), 3.60–3.88 (m, 211.2H, −CH_2_–O–CH_2_–CH_2_–O-PEGM and ILMC, OCH_2_CH_2_N ILMC, OCH_2_CH_2_N ILMC and CH_2_-2,5 ILMC), 3.45 (s, 17.4H, −O–CH_3_ PEGM), 3.23 (s, 3H, −N–CH_3_), 2.94–2.97 (t, 16.7H, CH_2_-Ph PhEtM), 2.33–2.35 (m, 4H, CH_2_-3,4 ILMC), 0.78–1.95 ((m, 75.7H, −CH_2_–C(CH_3_)) ILMC, PEGM and PhEtM, −CH_2_–C(CH_3_) ILMC, PEGM and PhEtM) ppm; ^19^F NMR (376.5 MHz, CDCl_3_): δ = −78.72 (s, CF_3_) ppm; IR (ATR mode): 3033 (w, ν_C–H,Ar_), 2875 (vs, ν_C–H,Alk_), 1729 (vs, ν_C=O_), 1455 (m, ν_C=C,Ar_), 1353 (m, ν_asSO_2__), 1246 (m), 1190 (s, ν_CF_), 1138 (vs, ν_O=C–O–_), 1111 (vs, ν_–C–O–C–_), 953 (w), 851 (w), 748 (w, δ_C–H,Ar_), 701 (w, δ_C–H,Ar_) cm^–1^; *M*_n(SEC)_ = 55.8 kDa, *M*_w_/*M*_n_ = 1.30; *M*_n(NMR)_ = 59.3 kDa.

#### 2.4.2 Poly[(ILMC_24_*-r-*PEGM_40_)-*b*-(PhEtM)_95_]

Yield: 0.61 g (64.0%); Conversion of PhEtM determined by ^1^H NMR: *q* = 0.59; ^1^H NMR (400.1 MHz, CDCl_3_): δ = 7.23–7.36 (m, 20.4H, CH_2_-Ph PhEtM), 4.14–4.22 (m, 13.5H, −CO–O–CH_2_ ILMC, PEGM and PhEtM), 3.60–3.88 (m, 71.1H, −CH_2_–O–CH_2_–CH_2_–O-PEGM and ILMC, OCH_2_CH_2_N ILMC, OCH_2_CH_2_N ILMC, CH_2_-2,5 ILMC), 3.45 (s, 5H, −O–CH_3_ PEGM), 3.21 (s, 3H, −N–CH_3_), 2.94–2.98 (t, 12.1H, CH_2_-Ph PhEtM) 2.33–2.34 ((m, 4H, CH_2_-3,4 ILMC), 0.78–1.46 (m, 33.4H, −CH_2_–C(CH_3_)) ILMC, PEGM and PhEtM, −CH_2_–C(CH_3_) ILMC, PEGM and PhEtM) ppm; ^19^F NMR (376.5 MHz, CDCl_3_): δ = −78.78 (s, CF_3_) ppm; IR (ATR mode): 3033 (w, ν_C–H,Ar_), 2892 (s, ν_C–H,Alk_), 1727 (vs, ν_C=O_), 1454 (m, ν_C=C, Ar_), 1353 (m, ν_asSO_2__), 1238 (m), 1191 (vs, ν_CF_), 1141 (vs, ν_O=C–O–_), 1112 (vs, ν_–C–O–C–_), 1060 (m), 953 (m), 852 (w), 747 (m, δ_C–H,Ar_), 700 (m, δ_C–H,Ar_), 616 (m), 571 (m), 511 (m) cm^–1^; *M*_n(SEC)_ = 27.8 kDa, *M*_w_/*M*_n_ = 1.20; *M*_n(NMR)_ = 51.8 kDa.

#### 2.4.3 Poly[(ILMA_12_*-r-*PEGM_68_)-*b*-(PhEtM)_96_]

Yield: 8.00 g (63.0%); calcd for (C_2908_H_4648_F_36_N_36_O_1012_S_24_): C, 33.27%; H, 4.19%; found: C, 33.06%; H, 4.11%; conversion of PhEtM determined by ^1^H NMR: *q* = 0.68; ^1^H NMR (600.2 MHz, DMSO-*d*_6_): δ = 9.09 (s, 0.9H, −N=CH–N), 7.76 (s, 0.9H, −(CH_3_)N–CH=CH–N(CH_2_)), 7.69 (s, 0.9H, −(CH_3_)N–CH=CH–N(CH_2_)), 7.19 (d, 37.0H, −CH_2_-Ph), 4.16 (t, *J= 7.2 Hz*, 3.1H, −CH_2_–N), 4.03 (br. s, 24.6H, −CO–O–CH_2_ PEGM, ILMC and PhEtM), 3.85 (s, 3.2H, −N(CH_3_)) 3.34–3.77 (m, 178.3H, −CH_2_–O–CH_2_–CH_2_–O), 3.24 (s, 16.6H, −O–CH_3_), 3.00 (s, 2H, −CH_2_–SO_2_), 2.82 (s, 14.2H, −CH_2_-Ph), 1.30–2.20 (m, 27.4H: −CH_2_–CH_2_–SO_2_, −N–CH_2_–CH_2_–CH_2_, −CH_2_–C(CH_3_)), 1.26 (sext, 3.0H, *J= 7.2 Hz*, −C–CH_2_–CH_2_–CH_3_), 0.55–1.23 (m, 45.0H, −CH_2_–C–CH_2_–CH_3_, −CH_2_–C(CH_3_)) ppm; ^19^F NMR (564.7 MHz, DMSO-*d*_6_): δ = −79.83 (s, CF_3_) ppm; IR (ATR mode): 2868 (s, ν_C–H,Alk_), 1724 (s, ν_C=O_), 1575 (w), 1454 (m, ν_C=C,Ar_), 1387 (w), 1349 (w), 1323 (m, ν_asSO_2__), 1267 (m), 1243 (m), 1224 (m, ν_CF_), 1177 (s, ν_SO_2__), 1102 (vs, ν_–C–O–C_), 948 (m), 851 (m), 748 (m, δ_C–H,Ar_), 699 (m, δ_C–H,Ar_) cm^–1^; *M*_n(SEC)_ = 53.2 kDa, *M*_w_/*M*_n_ = 1.49; *M*_n(NMR)_ = 58.3 kDa.

NMR spectra were recorded on AMX-400 or AMX-600 spectrometers (Bruker, Germany) at 25 °C in the indicated deuterated solvent and are listed in ppm. The signals corresponding to the residual protons and carbons of the deuterated solvent were used as an internal standard for ^1^H and ^13^C NMR, respectively. The C_6_F_6_ (−164.9 ppm) was utilized as an external standard for ^19^F NMR.

IR spectra were acquired on a Tensor 27 (Brucker, Germany) Fourier IR spectrometer using ATR technology (128 scans, resolution is 2 cm^–1^) and Spectragryph optical spectroscopy software.^36^

Kinematic viscosities (ν) of ILMs were measured using a calibrated CUSMC-300 Cannon-Ubbelohde semi-microcapillary viscometer at 25.0 °C. The dynamic viscosity (η) was calculated using the equation
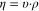


where ρ is the density of ILMs determined at 25.0 °C using a calibrated pycnometer.

Size exclusion chromatography (SEC) was used to determine the number-average molecular weights (*M*_n(SEC)_) and *M*_w_/*M*_n_ ratios for random and block copolymers. The study was performed on a 1200 Infinity gel permeation chromatograph (Agilent Technologies) equipped with a PLgel 5 μm MIXED-D column (Agilent Technologies), PLgel 5 μm (Agilent Technologies) precolumn, and an integrated refractive index detector. The system was operated at 50 °C and 1.0 mL/min flow using 0.1 M Li(CF_3_SO_2_)_2_N solution in DMF as an eluent. Poly(methyl methacrylate) standards (EasiVial PM, Agilent Technologies, *M*_p_ = 550–1558 × 10^3^) were used to perform calibration.

Atomic force microscopy (AFM) images were recorded with an MFP-3D Infinity microscope (Asylum Instruments/Oxford Instruments, United Kingdom) in tapping mode (30–35 °C, in air). AC160TS-R3 (Olympus, Japan) cantilevers were applied with a stiffness of 26 N m^–1^ and resonance frequency of 300 KHz. The images were recorded in the so-called “soft tapping mode,” to avoid deformation and indentation of the polymer surface by the tip. The domain periodicity was evaluated on an averaged power density spectrum (PSD) generated from a phase shift channel on three different 2 × 2 μm^2^ images. All of the images were collected with the maximum available number of pixels (512) in each direction. On each image, two profiles were taken, and for each, the distance over ten consecutive periods was recorded. The general procedure for the preparation of the samples for AFM was as follows: borosilicate glass coverslips (22 × 22 mm^2^, thickness no. 1 (0.13–0.16 mm), free from streaks, bubbles, and striations (Epredia, Netherlands)) from hydrolytic class I were rinsed with acetone, then with dichloromethane, and dried with air flow. The solution of block copolymer in anhydrous DMF with a concentration of 100 mg/mL was prepared at RT under an inert atmosphere. The solution was filtered through a 0.22 μm syringe filter and cast at 22 °C onto a glass coverslip placed on a leveled hotplate, whereupon the surface of the hotplate was heated to 80 °C. An inverted glass funnel with the neck filled with cotton was then placed over the top of the glass slide to ensure gradual evaporation (over the course of hours), thus enabling reorganization of the films to achieve (near-)equilibrium morphologies. Finally, the obtained films on the glass coverslips were transferred into the vacuum bell and dried at 80 °C/1 mbar for 24 h. Prior to AFM analysis, the sample surface was quickly rinsed with anhydrous ethanol for a few seconds and was then dried under a nitrogen flux. For additional AFM experiments (Figure S11), the glass coverslips were coated with golden leaves, rinsed with acetone, then with dichloromethane, and dried with air flow. The block copolymer solution was cast directly on the golden surface.

Thermal mechanical analysis (TMA) of block copolymer samples was performed under inert atmosphere (He) using a DIL 402 select Expedis dilatometer (NETZSCH, Germany) with a constant load of 0.3 N at a heating rate of 5 °C min^–1^ in the range of −100 to 150 °C. The heat distortion temperature (*T*_HDT_) was determined as a temperature at which a noticeable deformation under applied load and scanning/heating rate was observed.

Differential scanning calorimetry (DSC) of **poly(ILMC**_**12**_***-r-*****PEGM**_**68**_**)**, **poly(ILMC**_**24**_***-r-*****PEGM**_**40**_**)**, and **poly(ILMA**_**12**_***-r-*****PEGM**_**68**_**)** was performed on a DSC3+ STARe System differential calorimeter (Mettler Toledo, Switzerland) in the range of −80 to 150 °C at a heating rate of 5 °C min^–1^ under an argon atmosphere. When studying the **ILMA** and **ILMC**, a special low heating rate of 2 °C min^–1^ was applied as recommended for the investigation of viscous ionic liquids.^37^ Two heating–cooling cycles were carried out for each sample. Glass transition temperatures (*T*_g_) were calculated from the second heating curve.

Thermogravimetric analysis (TGA) was carried out in air on a TGA2 STARe System (Mettler Toledo, Switzerland), applying a heating rate of 5 °C min^–1^. The onset weight loss temperature (*T*_onset_) was determined as the point in the TGA curve at which a significant deviation from the horizontal was observed. The resulting temperature was then rounded to the nearest 1 °C.

Rheology measurements were performed using a Physica MCR 302 rheometer (Anton Paar, Austria) equipped with a CTD 450 temperature control device with a disposable aluminum plate–plate (diameter: 25 mm, measure gap: 1 mm) geometry. Measurements were recorded in the oscillation mode at an imposed 1% strain amplitude (γ), ensuring that both moduli *G*′ and *G*″ were obtained in the linear viscoelastic regime. All measurements were carried out at 25, 50, and 70 °C. Tests were repeated at least twice to insure good repeatability of the results.

Electrochemical impedance spectroscopy (EIS) was applied to determine the ionic conductivity (σ_DC_) of block copolymers using a VSP potentiostat/galvanostat (Bio-Logic Science Instruments, France). To avoid any influence of moisture/humidity on the conductivity of polymer electrolytes, the latter were preliminary dried at 60 °C/1 mbar for 12 h in the B-585 oven (Buchi Glass Drying Oven, Switzerland) filled with P_2_O_5_ and were transferred under vacuum inside an argon-filled glovebox (MBRAUN MB-Labstar, H_2_O and O_2_ content < 0.5 ppm). Polymers were sandwiched between two stainless steel (SS-316) blocking electrodes. The distance between the electrodes was kept equal to 250 μm using a Teflon spacer ring with the inner area of 0.502 cm^2^. Symmetrical stainless steel/copolymer/stainless steel assembly was clamped into the 2032 coin cell and was later taken out from the glovebox. Cell impedance was measured at an open-circuit potential (OCV) by applying a 10 mV perturbation in the frequency range from 10^–2^ to 2 × 10^5^ Hz and in a temperature range from 20 to 100 °C. Temperature was controlled using the programmed M-53 oven (Binder, Germany), where cells were allowed to reach thermal equilibrium for at least 1 h before each test.

Cyclic voltammetry (CV) was used to determine the electrochemical stability window (ESW) of block copolymers at 25 °C. The ESW was studied under an argon atmosphere in a glovebox (MBRAUN MB-Labstar, H_2_O and O_2_ content < 0.5 ppm) at room temperature using a VSP potentiostat/galvanostat (Bio-Logic Science Instruments, France). The three-electrode cells were assembled by sandwiching the polymer sample between two Pt foils (used as working and counter electrodes) and a silver mesh (used as a pseudo-reference electrode) to form the following architecture: Pt/coPIL/Ag mesh/coPIL/Pt. The ECW test was performed by scanning at 5 mV s^–1^ from the open-circuit potential (OCV) toward positive or negative potentials.

The metal–insulator–metal (MIM) capacitors were assembled on 25 × 25 mm^2^ glass substrates (University Wafers). The substrates were cleaned prior to fabrication in an ultrasonic bath in four steps starting with soapy water, followed by DI water, acetone, and methanol (5 min each) and were dried using nitrogen flow. The bottom contacts (2 nm of chromium followed by 50 nm of gold) of the capacitors were deposited on the cleaned substrates by physical vapor deposition (PVD) at a base pressure of 10^–6^ Torr.

To fabricate the PIL thin films on top of the Cr–Au bottom contacts, each ionic block copolymer was dissolved in chloroform at a concentration of 100 mg mL^–1^ and the solutions were filtered using hydrophobic PTFE syringe filters with 0.45 μm pores. The solution (300 μL) was coated on each substrate (replicate devices) using a Laurell WS-650-23 spin coater at 2000 rpm and further annealed for 1 h at 80 °C (i.e., above the glass transition temperatures of both blocks *T*_g1_ and *T*_g2_). Finally, the top electrode was deposited by coating 50 nm of gold via PVD.

The testing of MIMs by EIS was performed on a PGSTAT204 potentiostat/galvanostat (Metrohm, Switzerland). A frequency range of 10^–2^–10^6^ Hz was used with an AC amplitude of 10 mV for potentiostatic impedance measurements. The measurements were conducted at 25 °C under ambient conditions. The thicknesses of the polymer films were determined with a Dektak XT profilometer (Bruker, Germany). The areal capacitance was obtained using eqs 1 and 2 presented below
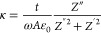
1
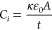
2where κ, the dielectric constant, is first calculated using the real (*Z*′) and imaginary (*Z*″) portions of the impedance plot, the thickness of the polyelectrolyte film (*t*), the angular frequency (ω), the capacitor surface area (*A*), and the permittivity of vacuum (ε_0_). Then, κ is used to calculate the capacitance (*C_i_*) of each individual capacitor on the substrates, and by dividing it by the surface area, the areal capacitance value is obtained.

The SWCNT-based TFTs were fabricated on 15 × 25 mm^2^ ultraflat quartz-coated glass substrates (purchased from Ossila, United Kingdom) starting with a cleaning procedure similar to MIM capacitors followed by the deposition of Cr–Au source–drain electrodes with the channel width and length of 1000 and 30 μm, respectively, using the same deposition recipe as MIM capacitors. The prepatterned substrates were then plasma-treated for 15 min and immersed in a 1% solution of octadecyltrichlorosilane (OTS) in toluene for 24 h at 70 °C to modify the surface with a hydrophobic self-assembled monolayer to improve SWCNT adhesion. The surface-modified substrates were rinsed with toluene to remove OTS solution and dried at 70 °C/0.1 mbar. The semiconducting layer was then deposited by drop-casting 0.2 μL of an ultrapure polymer-sorted SWCNT dispersion on the channel for every individual transistor (20 devices on each chip). The polymer excess was rinsed off the chips with 4 mL of toluene and the substrates were dried with a N_2_ stream and baked for 1 h at 200 °C in air to achieve a uniform monolayer of SWCNTs and to dry any leftover moisture.^31^ Next, the chloroform solutions of PILs were spin-coated on each chip and annealed under vacuum for 1 h at 80 °C (i.e., above the glass transition temperatures of both blocks *T*_g1_ and *T*_g2_). Finally, the devices were transferred into the glovebox and the gate electrodes were deposited onto them by the PVD process (50 nm of gold).

The TFTs were characterized using a Keithley 2614B (Tektronix) source-measure unit and a probe station to apply the source-gate (*V*_GS_) and source–drain (*V*_SD_) potentials. The output and transfer curves were recorded at 10 Hz to ensure the electrical double layer can be fully formed and all measurements were conducted in ambient conditions. The source–drain current (*I*_SD_) was modeled to calculate the threshold voltage (*V*_T_) (eq 3), charge mobility (μ) (eq 4), and transconductance (*g*_m_) (eq 5) in the linear region using the following equations
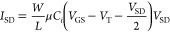
3
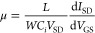
4
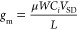
5In these equations, *W* and *L* stand for channel width (1000 μm) and channel length (30 μm), respectively. All channel widths and lengths were measured under an optical microscope and the mobility and transconductance values were corrected to eliminate electrode deposition shadowing effects from the calculations.

The transistors were characterized by sweeping the *V*_GS_ in pulsing frequencies with 20 ms on time and 80 ms off time at each voltage point. This mode has been shown to reduce hysteresis and allows polar and ionic materials to reach a well-polarized state. The frequency (*F*) reported was calculated using eq 6 presented below
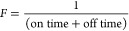
6

## 3 Results and Discussion

### 3.1 Design and Synthesis of Ionic Liquid-Like Monomers (**ILMA** and **ILMC**)

**Scheme 1:**
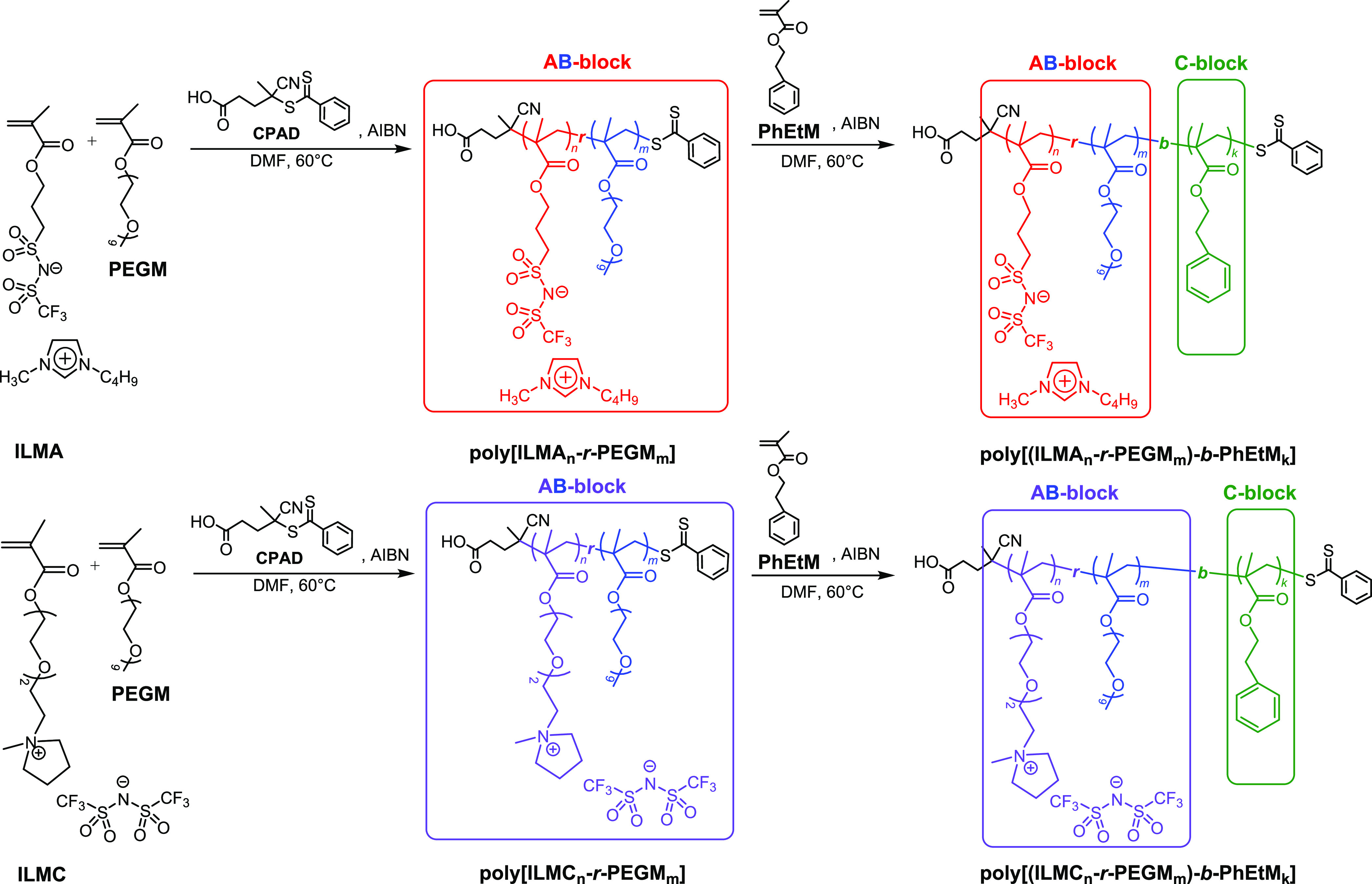
**Poly[(ILM*_n_-r-*PEGM*_m_*)-*b*-PhEtM*_k_*]** Block Copolymers Prepared in This Work via RAFT Copolymerization

High ionic conductivity is one of the key requirements for the proper operation of solid-state electrochemical devices, typically requiring a minimum conductivity of 10^–6^ S cm^–1^ at 25 °C.^1−4^ For this work, the cationic **ILMC** monomer previously synthesized by our group^34,35^ has been selected due to the high ionic conductivity it imparts to the polymeric materials generated from it. An equivalent anionic monomer **ILMA** (Scheme 1) was developed through an ion exchange reaction between lithium 1-[3-(methacryloyloxy)propylsulfonyl]-1-(trifluoromethanesulfonyl) imide^32^ and 1-butyl-3-methylimidazolium bromide. By analogy with **ILMC**, the chemically bonded delocalized TFSI anion in **ILMA** is separated from the methacrylic group by a flexible alkyl chain. In contrast with **ILMC**, **ILMA** possesses mobile imidazolium cations known to impart one of the highest conductivities measured for both ionic liquids and PILs.^9^ The structure and purity of both **ILMC** and **ILMA** were confirmed by NMR and IR spectroscopy and elemental analysis. Both monomers were viscous yellow liquids at r.t. with viscosities of 160 and 504 cP at 25 °C for **ILMC** and **ILMA**, respectively. DSC analyses revealed no crystallization or melting transitions on either heating or cooling, even at rates as low as 2 °C min^–1^. Via the same analyses, the glass transition temperatures (*T*_g_) were determined as −67 and −63 °C for **ILMC** and **ILMA**, respectively. Finally, the ionic conductivity of the monomers exceeded 10^–4^ S cm^–1^ at 25 °C (5.5 × 10^–4^ and 2.4 × 10^–4^ S cm^–1^ for **ILMC** and **ILMA**, respectively).

### 3.2 Poly(ILM-*r*-PEGM) Random Copolymers

**ILMC** and **ILMA** were further copolymerized with PEGM using the RAFT process to obtain the ionic block AB (Scheme 1). PEGM was chosen for its ability to drop the *T*_g_ and promote ion solubility leading to improved ionic conductivity. The optimized conditions involving 4-cyano-4-(phenylcarbonothioylthio)pentanoic acid (CPAD) as the chain-transfer agent and 2,2′-azobisisobutyronitrile (AIBN) as the initiator were applied for the precise control of the number-average molar masses (*M*_n_) and *M*_w_/*M*_n_ for poly(ILM-*r*-PEGM) copolymers.^27,32^ We selected a target *M*_n_ of ∼39–47 kDa and a monomer mol ratio of 1:5 mol to mimic our recent work on copolymerization of lithium derivatives of ILMA with PEGM,^27^ which were determined as the optimal parameters to achieve the highest ionic conductivity (Table 1). Similarly, for copolymerization of **ILMC** with PEGM, the optimal molar ratio (1:2) was taken from ref (38). However, to ensure the correct comparison between poly(ILMC-r-PEGM) and poly(ILMA-r-PEGM), an additional **poly(ILMC**_**12**_***-r-*****PEGM**_**68**_**)** copolymer having the 1:5.7 molar ratio between **ILMC** with PEGM units was also synthesized for comparison (Table 1). The *M*_n_ values of the obtained copolymers were determined by size exclusion chromatography (*M*_n(SEC)_) and NMR (*M*_n(NMR)_) using eqs S1–S3. While the determined *M*_n(NMR)_ values for all three random copolymers varied in the range of 33.6–40.8 kDa and were in a good agreement with theoretically calculated *M*_n_, the *M*_n(SEC)_ values showed deviations (Table 4S). The higher content of **ILMC** led to greater deviation of *M*_n(SEC)_ from *M*_n(NMR)_ and from theoretically calculated *M*_n_, which is likely explained by insufficient screening of the highly charged **poly(ILMC**_**24**_***-r-*****PEGM**_**40**_**)** macromolecules by (0.1 M) LiTFSI in the SEC eluent. It is important to note that due to the intense pink color of the CPAD RAFT agent, the obtained copolymers received the rose-pink or salmon-pink color as well. The chemical structure and purity of the obtained ionic poly(ILM-*r*-PEGM) copolymers were confirmed by ^1^H, ^19^F NMR, FTIR, and elemental analysis that can be found in Figures S1–S3. All three copolymers displayed *T*_g_ values (measured via DSC) in the range between −54 and −37 °C (Table S4). As expected, the copolymerization of **ILMA** and **ILMC** with PEGM (*T*_g poly(PEGM)_ = −62 °C) led to the decrease in the *T*_g_ values of the poly(ILM-*r*-PEGM) copolymers as compared to the corresponding poly(ILMA) and poly(ILMC) homopolymers.^32,38,39^ The presence of a single *T*_g_ in the DSC curves further suggests the formation of random copolymers. **Poly(ILMA**_**12**_***-r-*****PEGM**_**68**_**)** and **poly(ILMC**_**12**_***-r-*****PEGM**_**68**_**)** random copolymers demonstrated an ionic conductivity of 10^–6^ S cm^–1^ (25 °C), while **poly(ILMC**_**24**_***-r-*****PEGM**_**40**_**)** exhibited the highest conductivity of 1.6 × 10^–5^ S cm^–1^ at 25 °C, which is among the best performing PILs reported to date (Table S1). It is also interesting to note the difference in conductivity values obtained in this work for p**oly(ILMA**_**12**_***-r-*****PEGM**_**68**_**)** bearing a 1-butyl-3-methylimidazolium cation (7.6 × 10^–6^ S cm^–1^ at 25 °C) and for a similar copolymer with lithium counter cation (4.1 × 10^–7^ S cm^–1^ at 25 °C) reported by our team previously.^27^ Such difference in conductivity can be explained by the fact that the conductivity of polyanions is dependent on the counter cation used.^40,41^ While Li^+^ is known to be one of the “slowest” cations to move,^42^ the imidazolium cation has proved to impart one of the highest ionic conductivities when used in ionic liquids.^43^ Thus, the change from lithium to the imidazolium cation will significantly change the conductivity of PIL. Although all three poly(ILM-r-PEGM) copolymers were capable to demonstrate high ionic conductivity, they represent cold flowing liquids at 25 °C, rendering their integration into solid-state capacitors and thin-film transistors impossible.

**Table 1:** Selected Properties of **Poly[(ILM*_n_-r-*PEGM*_m_*)-*b*-PhEtM*_k_*]** Block Copolymers

block copolymer	PEGM/ ILM^a^	*M*_AB(NMR)_/*M*_C(NMR)_	*M*_n(NMR)_ (kDa)^b^	*M*_n (SEC)_ (kDa)^c^	*M*_w_/*M*_n_^c^_(SEC)_	*T*_g_s (°C)^d^	*T*_HDT_ (°C)^d^	*T*_onset_ (°C)^e^	σ_DC_, (S cm^–1^) at 25 °C	ESW (V)^f^	domain length (nm)^g^
**poly[(ILMA**_**12**_***-r-*****PEGM**_**68**_**)-*****b*****-PhEtM**_**96**_**]**	5.8	2.2:1	58.3	53.2	1.49	–55, 34	92	180	1.8 × 10^–6^	3.4	L, 36.8 ± 1.4
**poly[(ILMC**_**24**_***-r-*****PEGM**_**40**_**)-*****b*****-PhEtM**_**95**_**]**	1.7	1.9:1	51.8	27.8	1.20	–55, 40	125	210	2.9 × 10^–6^		L, 23.6 ± 0.8
**poly[(ILMC**_**12**_***-r-*****PEGM**_**68**_**)-*****b*****-PhEtM**_**97**_**]**	5.7	2.2:1	59.3	55.8	1.30	–61, 36	71	210	3.6 × 10^–6^	3.3	L, 40.1 ± 2.1

a By H^1^ NMR.

b Defined by eq S12.

c By GPC in 0.1 M solution of LiTFSI in DMF at 50 °C with PMMA standards.

d By TMA.

e Onset loss temperature by TGA.

f Electrochemical stability window (ESW) at 25 °C vs Ag^+^/Ag.

g By AFM on block copolymer-coatings, L - lamellar.

### 3.3 Block Copolymers Poly[(ILM*-r-*PEGM)-*b*-PhEtM]

#### 3.3.1 Synthesis

Poly(ILM-r-PEGM) copolymers were further used as macro-chain-transfer agents (macro-CTAs) in RAFT copolymerization with a PhEtM monomer (Scheme 1) applying the previously optimized conditions.^27^ The PhEtM monomer was chosen due to synthetic compatibility of the methacrylic functional group being similar to **ILMA**, **ILMC**, and PEGM, which is well-suited for the particular CPAD RAFT agent; and the existence of the aromatic moiety, which is known to be incompatible with ionic compounds leading to good phase separation of the resulting block copolymers.^18,20,28^ In our previous study using block copolymers with Li^+^ cations, it was found that lamellar morphology occurred only when high molecular weight block copolymers (*M*_n_ = 50–90 kDa) were targeted with the ion-containing block (AB) being at least twice as large as the neutral blocks (C) by weight and with a ratio of PEGM/ILM of approximately 5:1 by mol.^27^ Therefore, three poly[(ILM*-r-*PEGM)-*b*-PhEtM] block copolymers were synthesized with *M*_n(NMR)_ in the range of 51.8–59.3 kDa and with *M*_AB_/*M*_C_ ratio equal to 1.9–2.2 (Table 1 and Scheme 1). Poly[(ILM*-r-*PEGM)-*b*-PhEtM] were characterized by larger *M*_n(SEC)_ values and similar *M*_*w*_*/M*_*n*_ values (∼1.3–1.5) as compared to the parent poly(ILM-*r*-PEGM) ionic blocks, strongly supporting the formation of the desired block copolymers as sole products (Figure S4 and Table 1). The composition, purity, and chemical structure of the obtained block copolymers were supported by ^1^H, ^19^F NMR, FTIR spectroscopy, and elemental analysis and can be found in Figures S5 and S6.

#### 3.3.2 Thermal and Viscoelastic (Mechanical) Properties

**Figure 2:**
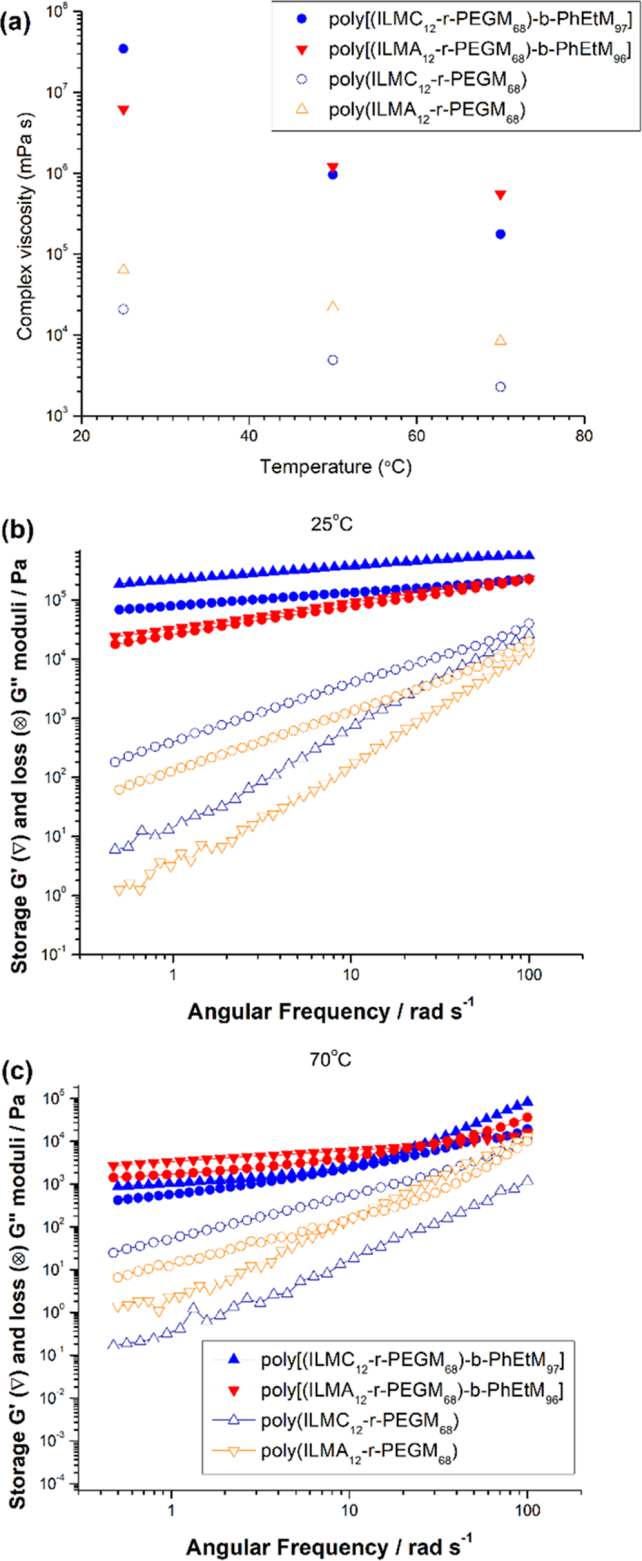
Temperature dependence of the complex viscosity (a) and frequency dependence of the storage modulus *G*′ (triangles) and the loss modulus *G*″ (circles) obtained at 25 °C (b) and 70 °C (c) for **poly[(ILMC**_**12**_***-r-*****PEGM**_**68**_**)-*****b*****-PhEtM**_**97**_**]**, **poly[(ILMA**_**12**_***-r-*****PEGM**_**68**_**)-*****b*****-PhEtM**_**96**_**]** (full symbols) and **poly(ILMC**_**12**_***-r-*****PEGM**_**68**_**), poly(ILMA**_**12**_***-r-*****PEGM**_**68**_**)** (open symbols).

Thermal properties of poly[(ILM*-r-*PEGM)-*b*-PhEtM] block copolymers were assessed by thermal mechanical (TMA) and thermogravimetric analyses (TGA) (Table 1 and Figure S7). TMA curves revealed the presence of the three distinct transition temperatures for all poly[(ILM*-r-*PEGM)-*b*-PhEtM] block copolymers. The first transition (*T*_g1_) was found to be in the low-temperature region, was attributed to the *T*_g_ of the ionic block, and was nearly coinciding with the *T*_g_ of the parent ionic poly(ILM-*r*-PEGM) random copolymer (Table S4). The second transition (*T*_g2_) located above room temperature was assigned to the *T*_g_ of the poly(PhEtM) block and varied depending on the ILM nature and PEGM/ILM ratio (Table 1). All block copolymers possessed the third transition, which was related to the heat distortion temperature (*T*_HDT_) at which a noticeable deformation (flow) was observed under applied load. The existence of two distinct *T*_g_ clearly demonstrated the presence of two segregated microphases in all poly[(ILM*-r-*PEGM)-*b*-PhEtM] block copolymers. The thermal degradation behavior of block copolymers was further studied via TGA. The weight loss profiles of block copolymers presented in Figure S7 revealed a one-step degradation mechanism. The *T*_onset_ values varied in a narrow range of 180–210 °C, lower vs the thermal stability of the neat poly(ILMA) and poly(ILMC) samples thanks to the inclusion of the less thermally stable poly(PEGM), which degrades at 160 °C (Table S4). In contrast, neat poly(ILMA) and poly(ILMC) show onset temperatures closer to 300 °C, consistent with TFSi-based polyelectrolytes.^9^ Interestingly, **poly[(ILMA**_**12**_***-r-*****PEGM**_**68**_**)-*****b*****-PhEtM**_**96**_**]** is less thermally stable than **poly[(ILMC**_**12**_***-r-*****PEGM**_**68**_**)-*****b*****-PhEtM**_**97**_**]**, suggesting that TFSI provides greater thermal stability as a counterion than incorporated into the polymer chain (Figure S7). Overall, the obtained PIL block copolymers were thermally stable to 180 °C in air, thus overcoming the majority of conventional liquid electrolytes with *T*_onset_ < 130 °C and making them particularly interesting for application in all-solid-state electrochemical devices. The viscoelastic properties of the PIL block copolymers were investigated by the dynamic rheological measurements (Figure 2). Using a plate–plate measuring system in a small amplitude oscillatory method at 25, 50, and 70 °C, the complex viscosity for **poly[(ILMA**_**12**_***-r-*****PEGM**_**68**_**)-*****b*****-PhEtM**_**96**_**]** and **poly[(ILMC**_**12**_***-r-*****PEGM**_**68**_**)-*****b*****-PhEtM**_**97**_**]** at a constant frequency of 1 Hz was found to decrease with temperature (Figure 2a). In comparison with the parent **poly(ILMA**_**12**_***-r-*****PEGM**_**68**_**)** and **poly(ILMC**_**12**_***-r-*****PEGM**_**68**_**)** random copolymers, the complex viscosity of **poly[(ILMA**_**12**_***-r-*****PEGM**_**68**_**)-*****b*****-PhEtM**_**96**_**]** and **poly[(ILMC**_**12**_***-r-*****PEGM**_**68**_**)-*****b*****-PhEtM**_**97**_**]** was significantly greater across all measurement frequencies, representing an improvement in application-relevant viscoelastic behavior following chain extension. Interestingly, the decrease in complex viscosity of **poly[(ILMA**_**12**_***-r-*****PEGM**_**68**_**)-*****b*****-PhEtM**_**96**_**]** followed the same slope as the decrease in complex viscosity for **poly(ILMA**_**12**_***-r-*****PEGM**_**68**_**)** and **poly(ILMC**_**12**_***-r-*****PEGM**_**68**_**)**, while the reduction for **poly[(ILMC**_**12**_***-r-*****PEGM**_**68**_**)-*****b*****-PhEtM**_**97**_**]** was more pronounced, implying a stronger temperature dependence (Figure 2a). The evolution of the storage (*G*′) and loss (*G*″) modulii as a function of the angular frequency at 25 and 70 °C are depicted in Figure 2b,c, respectively. At 25 °C, the *G*″ curves of **poly(ILMA**_**12**_***-r-*****PEGM**_**68**_**)** and **poly(ILMC**_**12**_***-r-*****PEGM**_**68**_**)** were above the *G*′ curves over the entire frequency range, indicating that these random copolymers were in a liquid or molten-like state. On the contrary, in the case of **poly[(ILMA**_**12**_***-r-*****PEGM**_**68**_**)-*****b*****-PhEtM**_**96**_**]** and **poly[(ILMC**_**12**_***-r-*****PEGM**_**68**_**)-*****b*****-PhEtM**_**97**_**]** block copolymers, the *G*′ curves were above the *G*″ curves over the full range of test frequencies, consistent with solid-like behavior. At 70 °C, differences in the behavior of **poly[(ILMA**_**12**_***-r-*****PEGM**_**68**_**)-*****b*****-PhEtM**_**96**_**]** and **poly[(ILMC**_**12**_***-r-*****PEGM**_**68**_**)-*****b*****-PhEtM**_**97**_**]** were found. The *G*′ curve of **poly[(ILMC**_**12**_***-r-*****PEGM**_**68**_**)-*****b*****-PhEtM**_**97**_**]** remained above the *G*″ curve over the entire frequency range, whereas in the case of **poly[(ILMA**_**12**_***-r-*****PEGM**_**68**_**)-*****b*****-PhEtM**_**96**_**]**, crossover is observed at 50 rad/s, with solid-like behavior at low frequencies and liquid-like behavior at higher frequencies. Additionally, the *G*′ and *G*″ curves for **poly[(ILMA**_**12**_***-r-*****PEGM**_**68**_**)-*****b*****-PhEtM**_**96**_**]** and **poly[(ILMC**_**12**_***-r-*****PEGM**_**68**_**)-*****b*****-PhEtM**_**97**_**]** were higher than those of the parent poly(ILM-*r*-PEGM) random copolymers at all measurement frequencies and temperatures, confirming significant improvements in the materials’ mechanical properties upon chain extension. Thus, all of the block copolymers show significantly improved moduli vs the parent ionic blocks. Currently, it is not feasible to make reasonable comparison with the other known linear PILs as very few groups reported to date the mechanical/rheological performance (Table S1) for their materials (likely because of the difficulties measuring such properties in liquid or low viscous cold flowing masses).

#### 3.3.3 Morphological Properties

**Figure 3:**
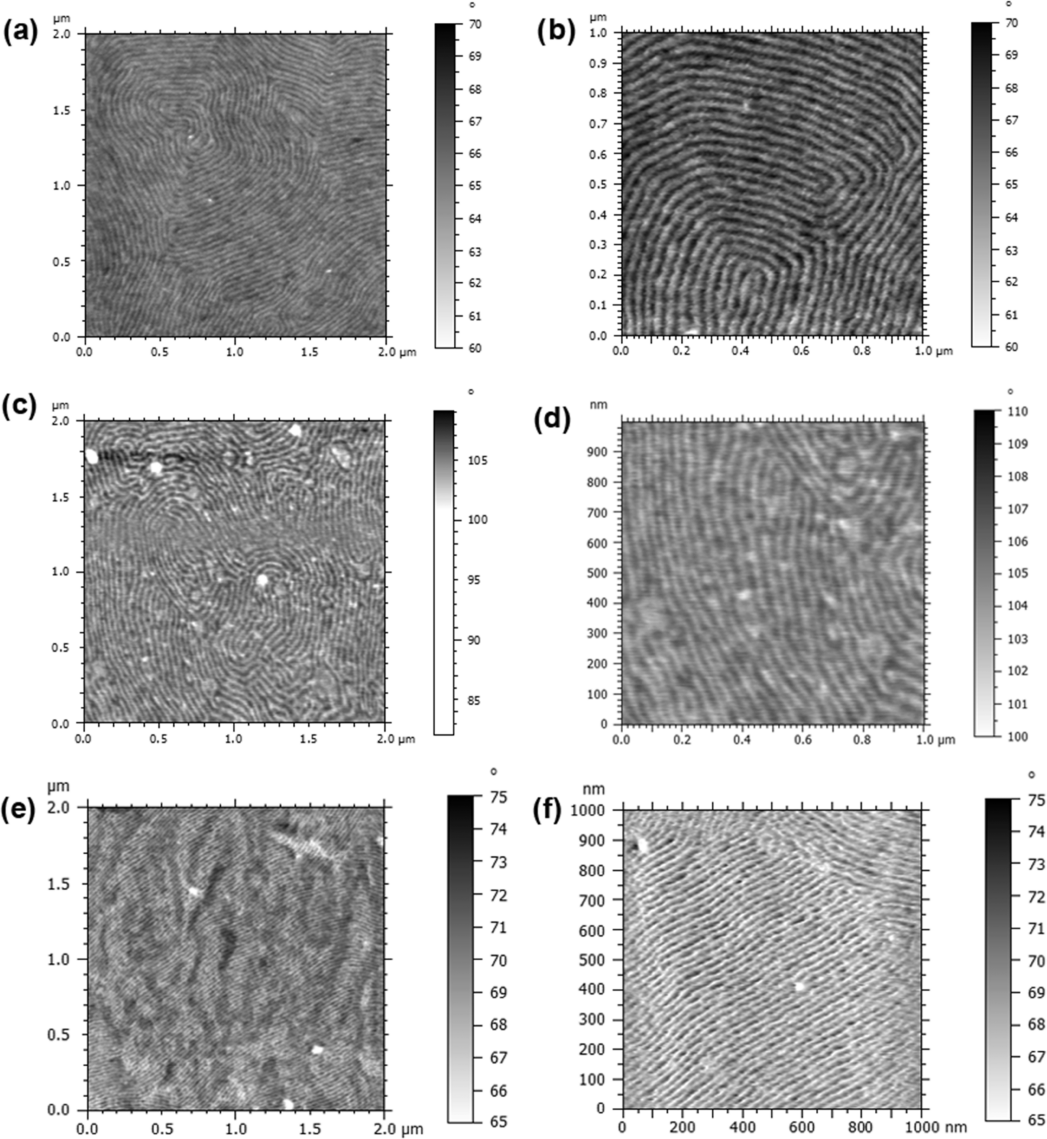
AFM images of **poly[(ILMA**_**12**_***-r-*****PEGM**_**68**_**)-*****b*****-PhEtM**_**96**_**]** (a, b), **poly[(ILMC**_**12**_***-r-*****PEGM**_**68**_**)-*****b*****-PhEtM**_**97**_**]** (c, d), and **poly[(ILMC**_**24**_***-r-*****PEGM**_**40**_**)-*****b*****-PhEtM**_**95**_**]** (e, f) films.

Recently, we have shown that the microphase-separation-driven self-assembly of copolymers consisting of ionic (conductive) and neutral (insulating) blocks at the semiconductor interface can provide improved TFT device performance.^29^ As such, one goal of the current work was to achieve similar levels of microphase separation in the copolymers produced here. To this, it was critical to first assess whether block copolymer synthesis was successful. This was confirmed through the observation of a single SEC peak (Figure S6) in combination with two distinct *T*_g_ values as detected via TMA. Furthermore, the measured ionic conductivities of the block copolymers were similar to those of the parent ionic blocks (Tables 1 and S4) despite the presence of a high content of neutral units in the former, implying microphase separation and the formation of continuous conductive channels. To visualize the associated block copolymer morphologies, thin films were cast from DMF solutions of **poly[(ILMA**_**12**_***-r-*****PEGM**_**68**_**)-*****b*****-PhEtM**_**96**_**]**, **poly[(ILMC**_**24**_***-r-*****PEGM**_**40**_**)-*****b*****-PhEtM**_**95**_**]**, and **poly[(ILMC**_**12**_***-r-*****PEGM**_**68**_**)-*****b*****-PhEtM**_**97**_**]**, dried at *T* > *T*_g2_ to enable polymer reorganization and the achievement of (near-) equilibrium morphologies, and explored by atomic force microscopy (AFM). AFM phase-contrast images (Figure 3) demonstrate that all three block copolymers exhibit lamellar morphologies with nanoscale domains in the range of 23–40 nm (Table 1). The domain size was independent of the nature of the ionic phase but changed with the ILM:PEGM ratio. The increase from 1:2 to 1:5 in the ILMC:PEGM ratio led to the increase in the domain size from ∼24 to ∼40 nm (Table 1). These results demonstrate the strong tendency for self-assembly in thin films of these PIL block copolymers, a favorable situation for TFT operation. Additionally, the AFM analysis was performed for **poly[(ILMA**_**12**_***-r-*****PEGM**_**68**_**)-*****b*****-PhEtM**_**96**_**]** films casted on a golden surface (Figure S11). Similar to films on glass coverslips, the films on the golden surface, mimicking the chromium-golden electrodes of MIMs and TFTs, showed lamellar phase separation, although with significantly low ordering.

#### 3.3.4 Electrochemical Properties

**Figure 4:**
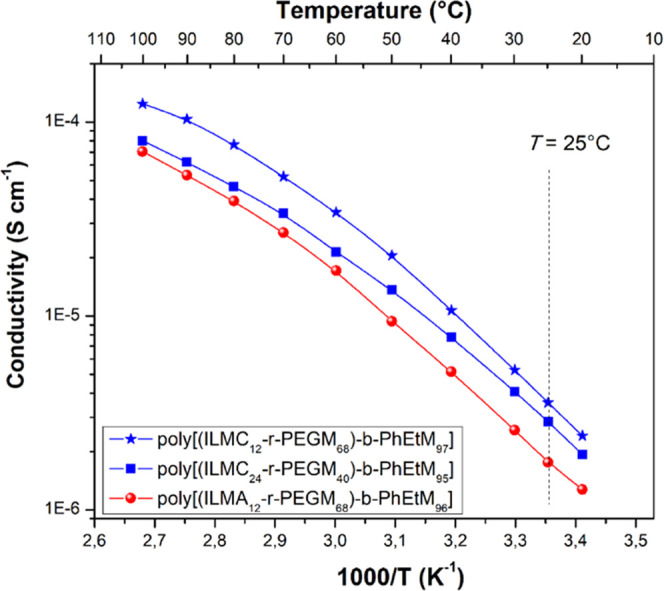
Ionic conductivity vs temperature dependence for **poly[(ILMA**_**12**_***-r-*****PEGM**_**68**_**)-*****b*****-PhEtM**_**96**_**]**, **poly[(ILMC**_**12**_***-r-*****PEGM**_**68**_**)-*****b*****-PhEtM**_**97**_**]**, and **poly[(ILMC**_**24**_***-r-*****PEGM**_**40**_**)-*****b*****-PhEtM**_**95**_**]**.

Ionic conductivity of poly[(ILM*-r-*PEGM)-*b*-PhEtM] block copolymers was measured as a function of temperature using electrochemical impedance spectroscopy. Figure 4 shows the conductivity of poly[(ILM*-r-*PEGM)-*b*-PhEtM] block copolymers as a function of inverse temperature between 20 and 100 °C. At 25 °C, the ionic conductivity for the three polymers varied between 1.8 × 10^–6^ and 3.6 × 10^–6^ S cm^–1^. Comparing cationic and anionic copolymers, it can be concluded that cationic **poly[(ILMC**_**12**_***-r-*****PEGM**_**68**_**)-*****b*****-PhEtM**_**97**_**]** showed slightly higher conductivity than its anionic analogue **poly[(ILMA**_**12**_***-r-*****PEGM**_**68**_**)-*****b*****-PhEtM**_**96**_**]** (Table 1). The increase in PEGM content also led to a slight increase in room-temperature ionic conductivity, as evident when comparing **poly[(ILMC**_**24**_***-r-*****PEGM**_**40**_**)-*****b*****-PhEtM**_**95**_**]** (2.9 × 10^–6^ S cm^–1^) and **poly[(ILMC**_**12**_***-r-*****PEGM**_**68**_**)-*****b*****-PhEtM**_**97**_**]** (3.6 × 10^–6^ S cm^–1^). In general, the ionic conductivities of the three block copolymers were very close to each other and nearly as high as those of the corresponding poly(ILM-*r*-PEGM) random copolymers (Tables 1 and S4), thus highlighting the benefits of existence of a continuous (percolated) conductive phase supported by more mechanically robust nonconductive blocks. We highlight here that, once percolation is achieved, the type (lamellar, cylindrical, etc.) and extent of orientation of the block copolymer microstructure become less important as far as the ion transport properties are concerned. Furthermore, the observed conductivity vs temperature plots slightly deviated from the ideal linear Arrhenius behavior at a temperature below 50 °C, while above 60 °C, the deviation became more pronounced (Figure 4). This suggests the diffusion of the mobile ions, namely, the 1-butyl-3-methylimidazolium cation in **poly[(ILMA**_**12**_***-r-*****PEGM**_**68**_**)-*****b*****-PhEtM**_**96**_**]** and the TFSI anion in **poly[(ILMC**_**24**_***-r-*****PEGM**_**40**_**)-*****b*****-PhEtM**_**95**_**]** and **poly[(ILMC**_**12**_***-r-*****PEGM**_**68**_**)-*****b*****-PhEtM**_**97**_**]**, occurred not only through the hopping mechanism between the chemically bonded ions but also is affected by local segmental motion of the oxyethylene fragments in the side dangling chains.^44^ To provide additional context for this analysis, ionic conductivities of 10^–11^–10^–7^ S cm^–1^ (25 °C) for PIL random copolymers^45,46^ and 10^–9^–10^–7^ S cm^–1^ (25 °C) for the block copolymers^28,45,47,48^ may be considered typical. In contrast, the copolymers reported here show significantly higher ionic conductivities that approach some of the highest values reported to date for PILs in general (Table S1). While the materials synthesized in this work do not represent the most conductive linear PILs ever reported (Table S1), it is nonetheless quite significant that they are among the top ten when considering conductivity at 25 °C. This alone already represents an important result given the relatively small rang of conductivities into which the performance of most of these materials falls (the majority of PILs showed σ = 1.0 × 10^–5^ to 6.7 × 10^–5^ S cm^–1^ at 25 °C). What sets the presented work apart is the fact that we combine such high levels of conductivity with good mechanical properties, without having to resort to the inclusion of any small molecules (such as ionic liquids) that could leak out or be extracted, leading to safety issues and loss of performance.

Further, on the investigation of poly[(ILM*-r-*PEGM)-*b*-PhEtM] block copolymers, the electrochemical stability window (ESW) was performed by cyclic voltammetry using the three electrodes scheme (Figure S8). The anodic and cathodic scans were studied separately on fresh samples. The oxidation potentials for **poly[(ILMA**_**12**_***-r-*****PEGM**_**68**_**)-*****b*****-PhEtM**_**96**_**]** and **poly[(ILMC**_**12**_***-r-*****PEGM**_**68**_**)-*****b*****-PhEtM**_**97**_**]** against the Pt electrodes were found to be 1.1 and 1.0 V vs Ag^+^/Ag, respectively, as indicated by the sudden increase of the anodic current (Figure S8). The reduction potentials were almost similar for both copolymers reaching −2.3 V vs Ag^+^/Ag. The total exhibited ESWs were found to be 3.4 and 3.3 V for **poly[(ILMA**_**12**_***-r-*****PEGM**_**68**_**)-*****b*****-PhEtM**_**96**_**]** and **poly[(ILMC**_**12**_***-r-*****PEGM**_**68**_**)-*****b*****-PhEtM**_**97**_**]**, respectively (Table 1), which were more than sufficient for stable operation of the SWCNT-based TFTs described here. The obtained ESW values were comparable with those reported previously for imidazolium (3.9 V vs Ag^+^/Ag^34^)-based PILs, although lower than for 1,2,3-triazolium PILs (5.2–5.7 V vs Ag^+^/Ag^49^) studied in similar experimental conditions. The majority of the ESW data published for anionic PILs was measured vs Li^+^/Li and varied in the range of 4.4–4.9 V.^50,51^

#### 3.3.5 Thin-Film Metal–Insulator–Metal (MIM) Capacitors

**Figure 5:**
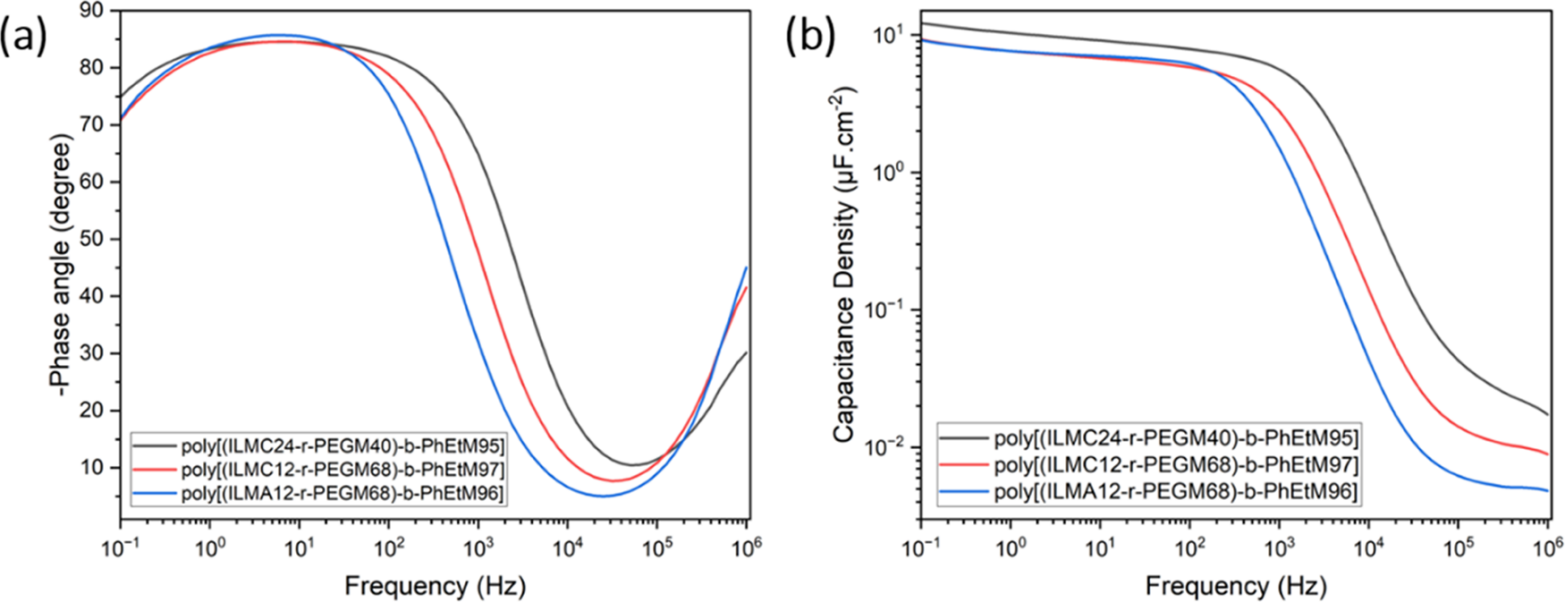
Phase angle (a) and areal capacitance (b) at different operating frequencies for poly[(ILM-r-PEGM)-b-PhEtM] block copolymers..

Poly[(ILM*-r-*PEGM)-*b*-PhEtM] block copolymers were then integrated into thin-film MIM capacitors and characterized by frequency-dependent impedance spectroscopy. The change in the phase angle (Figure 5) defines the diffusion of the ions toward the electrodes and provides critical information about the formation of an electrical double layer (EDL) in the materials. For example, as the frequency increases, the phase angle drops due to the inability of the ions to reach the electrode in time;^52^ this coincides with a significant decrease in capacitance. From Figure 5, poly[(ILM*-r-*PEGM)-*b*-PhEtM] block copolymers appear to experience the onset of EDL formation at frequencies of at least 10^6^ Hz. EDL formation appears to be complete at ∼10^3^ Hz in all cases, with completion occurring at slightly higher frequencies in the TFSI-containing block copolymers. This is slightly superior to the performance of the reported PIL block copolymers^28,47^ and significantly exceeds that of the reported random copolymers (which are solid at room temperature^45,48^). These results suggest the **poly[(ILMC**_**24**_***-r-*****PEGM**_**40**_**)-*****b*****-PhEtM**_**95**_**]** exhibits favorable EDL formation with a greater areal capacitance of 17.2 μF cm^–2^ compared to 9.0–9.7 μF cm^–2^ measured for **poly[(ILMA**_**12**_***-r-*****PEGM**_**68**_**)-*****b*****-PhEtM**_**96**_**]** and **poly[(ILMC**_**12**_***-r-*****PEGM**_**68**_**)-*****b*****-PhEtM**_**97**_**]** block copolymers. The superior areal capacitance value for **poly[(ILMC**_**24**_***-r-*****PEGM**_**40**_**)-*****b*****-PhEtM**_**95**_**]** is due to the higher ionic portion, with a PEGM/ILM ratio of 1.7 compared to the other two PILs that have a ratio of 5.7 and 5.8, respectively. A lower PEGM/ILM ratio increases the portion of the polymer structure that contributes to EDL formation and therefore leads to more efficient polarization of charges species. It is noteworthy that these values are significantly larger than all of those from our previous reports involving PIL block copolymers (0.2–2 μF cm^–2^).^28,45−48^ This improvement can be attributed to the decreased *T*_g_ for the current polymers compared to previous examples with a mechanical stable block/random units of methacrylates or styrene. A lower *T*_g_ facilitates the movement of mobile ions between polymer chain networks and leads to higher ionic conductivity, more efficient EDL formation, and higher capacitance values.

#### 3.3.6 Thin-Film Transistors (TFTs)

**Figure 6:**
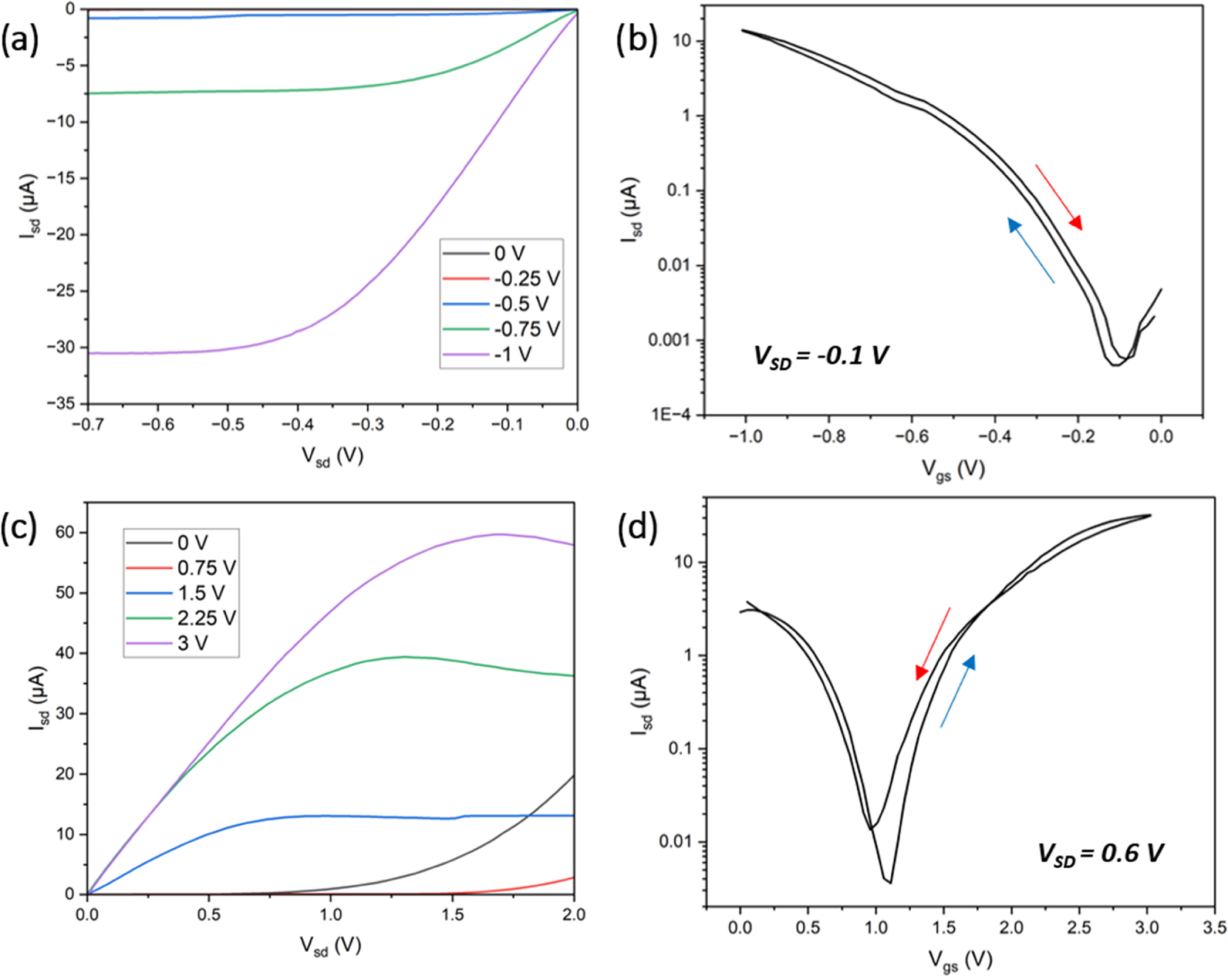
Output and transfer curves of **poly[(ILMC**_**24**_**-r-PEGM**_**40**_**)-b-PhEtM**_**95**_**]** tested under p-type (a, b) and **poly[(ILMA**_**12**_**-r-PEGM**_**68**_**)-b-PhEtM**_**96**_**]** under n-type (c, d) conditions. The blue and red arrows in panels (b) and (d) show the forward and backward sweeps, respectively, in the transfer curves.

Poly[(ILM*-r-*PEGM)-*b*-PhEtM] block copolymers were integrated into semiconductive single-walled carbon nanotubes (SWCNT) top gate bottom contact TFTs as the gating material. High ambipolar charge transport characteristics, good mechanical stability, and ease of processing make SWCNT ideal candidates for integration into TFTs.^30,53,54^ The characteristic transfer curves and output curves for the corresponding TFTs containing poly[(ILM*-r-*PEGM)-*b*-PhEtM] block copolymers are depicted in Figure 6 with the key parameters tabulated in Table 2. The transfer curves show clear ambipolar behavior of the SWCNT TFTs with low hysteresis (at 10 Hz) and low threshold voltage (<1.5 V). This may be explained by the high ionic conductivity of the poly[(ILM*-r-*PEGM)-*b*-PhEtM] block copolymers coupled with complete EDL formation. In contrast, in our work with an analogous TFT based on poly(2-(methacryloyloxy)ethyl trimethylammonium bis(trifluoromethylsulfonyl)azanide-*r*-methyl methacrylate) (poly(METATFSI-MMA)),^46^ the low conductivity of this PIL random copolymer (1.8 × 10^–9^ S cm^–1^ at 25 °C) led to significant hysteresis when operating at high frequencies.

**Table 2:** SWCNT Thin-Film Transistor Characteristics of **Poly[(ILMC_24_-*r*-PEGM_40_)-*b*-PhEtM_95_]** and **Poly[(ILMA_12_-*r*-PEGM_68_)-*b*-PhEtM_96_]** under p-Type and n-Type Conditions, Respectively

gating polymer material	μ (cm^2^/V·s)^a^	*I*_on/off_^b^	*V*_T_ (V)^c^	*H* (V)^d^	*g*_m_ (μS)^e^
**poly[(ILMC**_**24**_***-r-*****PEGM**_**40**_**)-*****b*****-PhEtM**_**95**_**]**	0.24 ± 0.11 (p-type)	10^4^	–0.36 ± 0.01	0.07 ± 0.03	22.8 ± 15.7
**poly[(ILMA**_**12**_***-r-*****PEGM**_**68**_**)-*****b*****-PhEtM**_**96**_**]**	0.02 ± 0.01 (n-type)	10^4^	1.10 ± 0.15	0.03 ± 0.02	4.9 ± 3.6

a Charge mobility.

b Ratio of source–drain on current versus off current.

c Threshold voltage.

d Device hysteresis.

e Transconductance value.

While **poly[(ILMC**_**24**_**-r-PEGM**_**40**_**)-b-PhEtM**_**95**_**]**, with a mobile anion, was tested under p-type conditions, the **poly[(ILMA**_**12**_**-r-PEGM**_**68**_**)-b-PhEtM**_**96**_**]** with a mobile cation was studied under n-type conditions. The former shows higher transconductance (Table 2) owing to a higher areal capacitance compared to the latter. This (p-type) characterization improves the output current^55^ but can slow down charge carrier transport and lead to lower switching speeds and mobility values. In softer polymers with a lower *T*_*g*_ and higher ion conductivities, the ions can more easily diffuse into and electrochemically dope the semiconductor, exhibiting a device behavior similar to electrochemical transistors. It has also been reported that integrating gate dielectrics with high capacitance densities can create polar interfaces, which introduce charge traps at the dielectric–semiconductor interface, hindering charge mobility.^52^ Electrochemical doping and more polar interfaces explain the reduced mobility for poly[(ILM-*r*-PEGM)-*b*-PhEtM] in this study compared to our previous study using poly(METATFSI-MMA). Doping in air (by water or oxygen) tends to enhance the p-type charge mobility of SWCNT transistors, while degrading the n-type charge transport,^56^ which is consistent with the higher mobility observed for **poly[(ILMC**_**24**_**-r-PEGM**_**40**_**)-b-PhEtM**_**95**_**]**. Despite relatively lower charge mobility values in this study, the transconductances obtained are in the same range as poly(METATFSI-MMA) due to the significantly higher areal capacitance of poly[(ILM-*r*-PEGM)-b-PhEtM]. Furthermore, the low threshold voltages (*V*_T_) of −0.3 and 1.1 V in respective p-type and n-type operation make these devices promising for low-power electronics.

## 4 Conclusions

We report the synthesis and characterization of well-defined poly[(ILM*-r-*PEGM)-*b*-PhEtM] cationic and anionic block copolymers for use in printed electronics. The block copolymers were found to readily self-assemble into a lamellar morphology, providing a mechanically robust domain and a low glass transition-based domain for the transport of ionic charges. These novel polymers were characterized by very high ionic conductivities and elevated capacitance values when integrated into thin-film parallel plate capacitors. The obtained values of the areal capacitance (up to 17.2 μF cm^–2^) can be named among the greatest capacitances reported in the literature for solid polymer electrolytes. The PIL block copolymers were used in the assembly of solid-state single-walled carbon-nanotube-based thin-film transistors providing low hysteresis, low threshold voltage (0.3 and 1.1 V for p-type and n-type operation), and apparent ambipolar behavior. The eminent conductivity of the PILs translated to high-frequency electrical double-layer formation that enables high-frequency operation of TFTs. These very positive results highlight the potential of this new family of block copolymers to enable the formation of high-performance thin-film electronics based on highly conductive solid-state polymeric materials amenable to high-speed printing.

## Supporting Information Available

Experimental details and loadings for the synthesis of poly(ILM*_n_-r-*PEGM*_m_*) random and **poly[(ILM_*n*_-*r-*PEGM*_m_*)-*b*-PhEtM*_k_*]** block copolymers; selected properties of poly(ILM*_n_*-*r-*PEGM*_m_*) random copolymers; description of the characterization techniques for the determination of monomers conversion (q), ILM:PEGM ratio, monomer units numbers, and *M*_n(NMR)_ for poly(ILM-*r*-PEGM); description of the characterization techniques for the determination of monomers conversion (*q*), PhEtM units number, and *M*_n(NMR)_ for block copolymers; GPC traces, TGA traces, and electrochemical stability windows of copolymers; and an overview of the most highly conductive PILs reported to date (PDF)
