# Supplementary material for: Mechanically Robust Poly(ionic liquid) Block Copolymers as Self-Assembling Gating Materials for Single-Walled Carbon-Nanotube-Based Thin-Film Transistors
Source: ACS Appl Polym Mater. 2023 Mar 30;5(4):2639–53. doi: 10.1021/acsapm.2c02223 (PMC10111415; doi:10.1021/acsapm.2c02223)
Supplement: Supplementary file 1 — ap2c02223_si_001.pdf [file ap2c02223_si_001.pdf]

Supporting information

Mechanically robust poly(ionic liquid) block copolymers as self-assembling gating material for single walled carbon nanotube based thin film transistors

Daniil R. Nosov[a,b,‡], Bahar Ronnasi[c,‡], Elena I. Lozinskaya[d], Denis O. Ponkratov[d], Laura Puchot[a], Patrick Grysan[a], Daniel F. Schmidt[a], Benoît H. Lessard[c,e,] and Alexander S. Shaplov[a,]**

[a]*Luxembourg Institute of Science and Technology (LIST), 5 avenue des Hauts-Fourneaux, L-4362 Esch-sur-Alzette, Luxembourg;*

[b]*Department of Physics and Materials Science, University of Luxembourg, 2 Avenue de l'Université, L-4365 Esch-sur-Alzette, Luxembourg*

[c]*Department of Chemical & Biological Engineering, University of Ottawa, 161 Louis Pasteur, Ottawa, ON, K1N 6N5, Canada;*

[d]*A.N. Nesmeyanov Institute of Organoelement Compounds Russian Academy of Sciences (INEOS RAS), Vavilov str. 28, bld. 1, 119334 Moscow, Russia*

[e]*School of Electrical Engineering and Computer Science, University of Ottawa, 800 King Edward Ave., Ottawa, ON, Canada*

Corresponding Authors: *alexander.shaplov@list.lu* (ASS) and *benoit.lessard@uottawa.ca (BHL)*

Table S1 An overview of the most highly conductive PILs reported to date (updated on September, 2022).

No	PIL	M_n[c], kDa	Ionic conductivity: σ_{DC}[a], S cm^{-1}	Ionic conductivity: T[b], °C	Physical state of PIL (mechanical properties)	Ref
1	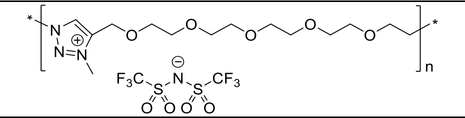	8.2[d]	1.2×10^{-4}	30	amber-colored low MM viscous material (n.d.)	1
2	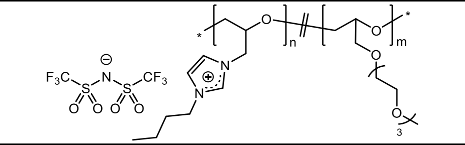	5.4	1.2×10^{-4}	25	cold flowing viscous mass (n.d.)[f]	2
3	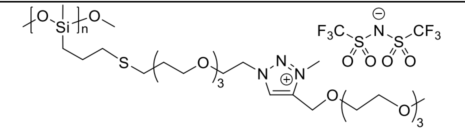	30.2	6.7×10^{-5}	30	yellow viscous liquid (n.d.)	3
4	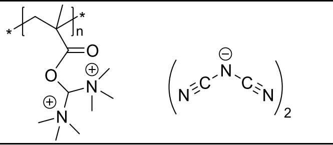	1830[d]	5.5×10^{-5}	25	colorless coatings on metals, brittle and opaque self-standing films (n.d.)	4
5	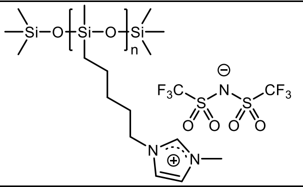	2.4[d]	5.0×10^{-5}	25	Brown oil (dynamic shear loss modulus G″ (rheology, -30oC): 130 MPa)	5
6	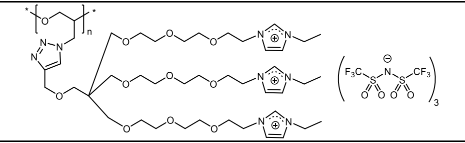	1900.0[d]	3.6×10^{-5}	25	cold flowing viscous mass (n.d.)	6
7	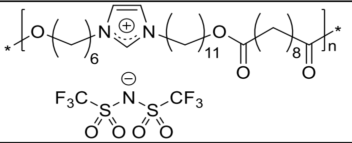	26	3.2×10^{-5}	25	clear viscous oil (n.d.)	7
8	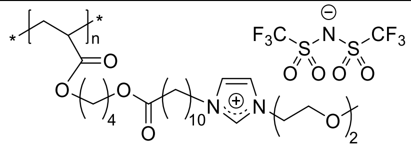	6.7	2.8×10^{-5}	25	(n.d.)	8
9	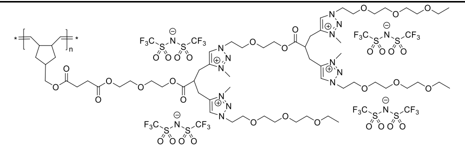	23.0	2.4×10^{-5}	30	from purple sticky soft solid to cross-linked soft self-standing films (maximum stress and the elongation at break (tensile testing, 25°C): 0.17 MPa and 170%)	9

10	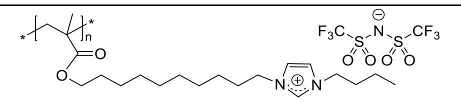	26.8	2.5×10^{-5}	30	yellow viscous oil (n.d.)	10
11	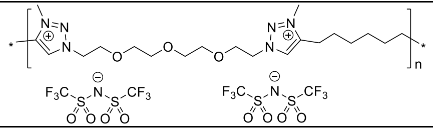	71.7	1.8×10^{-5}	25	brown gummy sticky solid (n.d.)	11
12	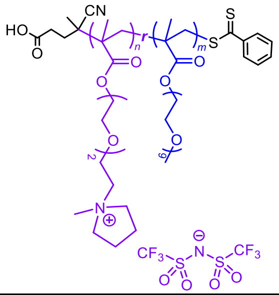	39.8	1.6×10^{-5}	25	**This work**	
13	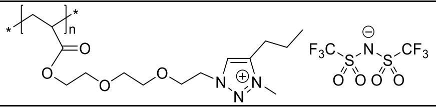	113.6	1.1×10^{-5}	30	orange viscous material (n.d.)	12
14	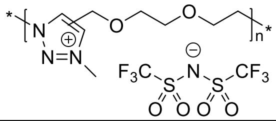	44	1.0×10^{-5}	25	dark brown viscous gummy solid (n.d.)	13
15	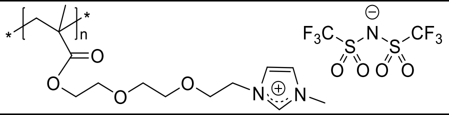	193.8	6.9×10^{-6}	25	transparent cold flowing viscous mass (n.d.)	14
16	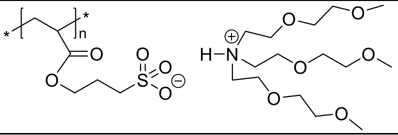	99.5	6.3×10^{-6}	27	(n.d.)	15
…	…	…	…	…		…
17	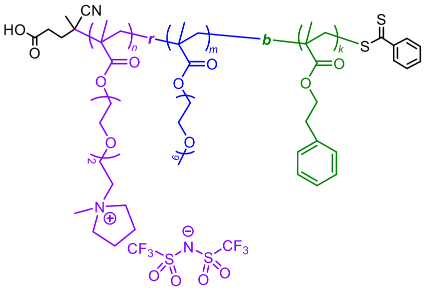 **poly[(ILMC$_{12}$-*r*-PEGM$_{68}$)-*b*-PhEtM$_{97}$]**	55.8	3.6×10^{-6}	25	**This work**	

[a] DC conductivity under anhydrous conditions;
[b] Temperature at which σ_{DC} was measured;
[c] Number-average molecular weight;
[d] Number-average molecular weight measured for neutral precursor;
[e] Weight-average molecular weight;
[f] N.d.=not determined.

Table S2 Loadings for the synthesis of random **poly(ILM$_n$-*r*-PEGM$_m$)** copolymers.

	[PEGM]		[ILM]		[CPAD]		[AIBN]		[DMF]		([PEGM]$_o$+[ILM]$_o$):[CPAD]$_o$:[AIBN]$_o$
	(g)	(mmol)	(g)	(mmol)	(mg)	(μmol)	(mg)	(μmol)	(g)	(mL)	
**poly(ILMC$_{12}$-*r*-PEGM$_{68}$)**	1.02	2.04	0.23	0.41	7.40	26.50	0.87	5.30	3.00	3.2	92.4:1:0.2
**poly(ILMC$_{24}$-*r*-PEGM$_{40}$)**	0.50	1.01	0.57	1.00	7.60	27.20	0.89	5.40	3.21	3.4	73.9:1:0.2
**poly(ILMA$_{12}$-*r*-PEGM$_{68}$)**	7.85	15.71	1.50	3.14	56.70	203.33	6.67	40.70	28.06	29.6	92.7:1:0.2

Table S3 Loadings for the synthesis of **poly[(ILM$_n$-*r*-PEGM$_m$)-*b*-PhEtM$_k$]** block copolymers.

	[PhEtM]		[macro-CTA]		[AIBN]		[DMF]		[PhEtM]$_o$:[macro-CTA]$_o$:[AIBN]$_o$
	(g)	(mmol)	(g)	(μmol)	(mg)	(μmol)	(g)	(mL)	
**poly[(ILMC$_{12}$-*r*-PEGM$_{68}$)-*b*-(PhEtM)$_{97}$]**	0.46	2.39	0.50	12.25	0.40	2.45	2.87	3.0	195:1:0.2
**poly[(ILMC$_{24}$-*r*-PEGM$_{40}$)-*b*-(PhEtM)$_{95}$]**	0.45	2.38	0.50	14.87	0.49	2.98	2.88	3.0	160:1:0.2
**poly[(ILMA$_{12}$-*r*-PEGM$_{68}$)-*b*-(PhEtM)$_{96}$]**	4.77	21.32	8.10	203.00	6.64	40.50	38.59	40.7	105:1:0.2

Table S4 Selected properties of **poly(ILM$_n$-*r*-PEGM$_m$)** random copolymers.

Random copolymer	PEGM: ILM [a]	M_{NMR} (κDa)[b]	$M_{n\,(SEC)}$ (κDa)[c]	M_w/M_n[c] (SEC)	M_{target} (κDa)	T_g, (°C)[d]	T_{onset}, (°C)[e]	σ_{DC}, (S/cm) at 25°C
**poly(ILMA$_{12}$-*r*-PEGM$_{68}$)**	5.8	39.8	39.4	1.35	47.4	-40	165	7.6×10^{-6}
**poly(ILMC$_{24}$-*r*-PEGM$_{40}$)**	1.7	33.6	21.3	1.10	39.6	-37	160	1.6×10^{-5}
**poly(ILMC$_{12}$-*r*-PEGM$_{68}$)**	5.7	40.8	29.6	1.11	46.3	-54	155	9.3×10^{-6}
**poly(PEGM)**[f]	-	-	23.6	1.16	25	-62	160	-
**poly(ILMA)**[f]	-	-	35.1	1.53	100	-13	275	6.5×10^{-7}
**poly(ILMC)**[f]	-	-	49.2	1.62	100	-8	315	6.1×10^{-6}
**poly(PhEtM)**[f]	-	-	17.1	1.18	37.5	48	200	-

[a] By ^{1}H NMR.
[b] Defined by equations eq. S2 and S3.
[c] By GPC in 0.1 M solution of LiTFSI in DMF at 50°C with PMMA standards.
[d] By DSC.
[e] Onset loss temperature by TGA.
[f] For comparison.

Determination of monomers conversion (q) in the synthesis of poly(ILM-r-PEGM) copolymers

For the calculation of total monomers conversion (q) the ^{1}H NMR spectra of the reaction mixture were recorded before and after polymerization. The solvent (DMF) was used as an internal standard as it was not evaporated in course of reaction due to its high boiling point. In ^{1}H NMR spectra of the reaction mixture before polymerization each of the integrals of $C{=}CH_2$ signals (a and a') from both monomers at 5.68-5.69 and 5.17-5.18 ppm were assigned as "1". At the same time the signal of DMF ($\underline{H}$-CO-$N(CH_3)_2$, c) at 7.58 ppm was integrated. Then, in the ^{1}H NMR spectra of the reaction mixture after polymerization the signal of DMF (Figure S1, c) was integrated and set as the standard with the same value as before. The $C{=}CH_2$ signals of the residual monomers (Figure S1, b and b') were integrated and the conversion (q) was calculated in accordance with the equation (eq. S1):

$$q = \frac{2-(b+b')}{2} \quad \text{(eq. S1)}$$

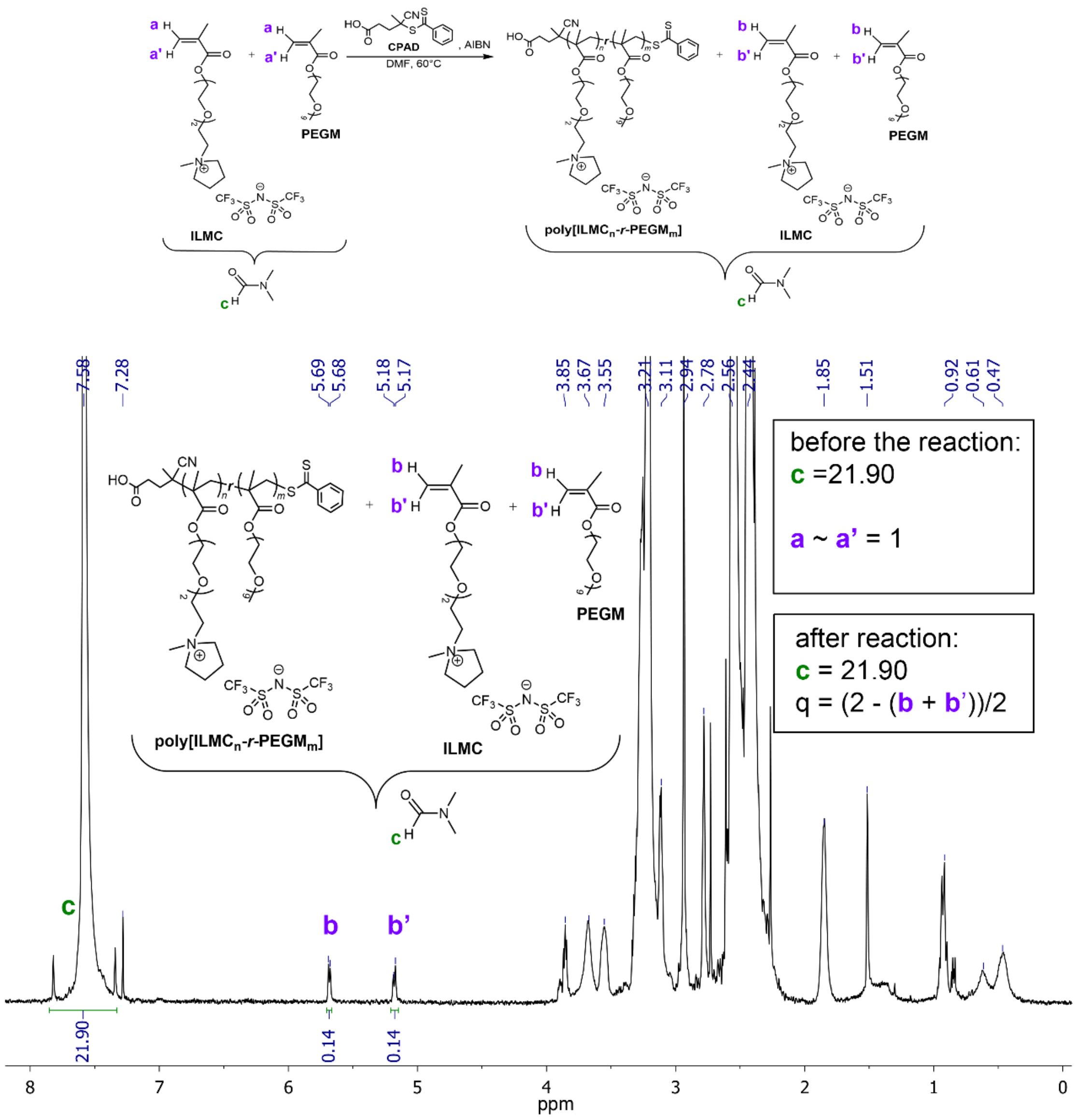

Figure S1. ^{1}H NMR of reaction mixture in $CDCl_3$ after the ILMC and PEGM random RAFT copolymerization (poly($ILMC_{24}$-*r*-$PEGM_{40}$) synthesis).

Determination of ILM:PEGM ratio in poly(ILM-r-PEGM) copolymers

The proportion of ILMC (X_{ILMC}) and PEGM (X_{PEGM}) units in poly(ILMC-*r*-PEGM) was determined by ^{1}H NMR of isolated copolymer sample using the integrals of the signals at 3.39 ppm (-O-C<u>H</u>3 from PEGM, see **l** on Figure S2) and 3.15 ppm (-N-C<u>H</u>3 from ILMC, see **i** on Figure S2) and the equations S2 and S3:

$X_{ILMC} = \frac{i}{i+l}$ (eq. S2),

$X_{PEGM} = \frac{l}{i+l}$ (eq. S3),

PEGM : ILMC = X_{PEGM} / X_{ILMC}

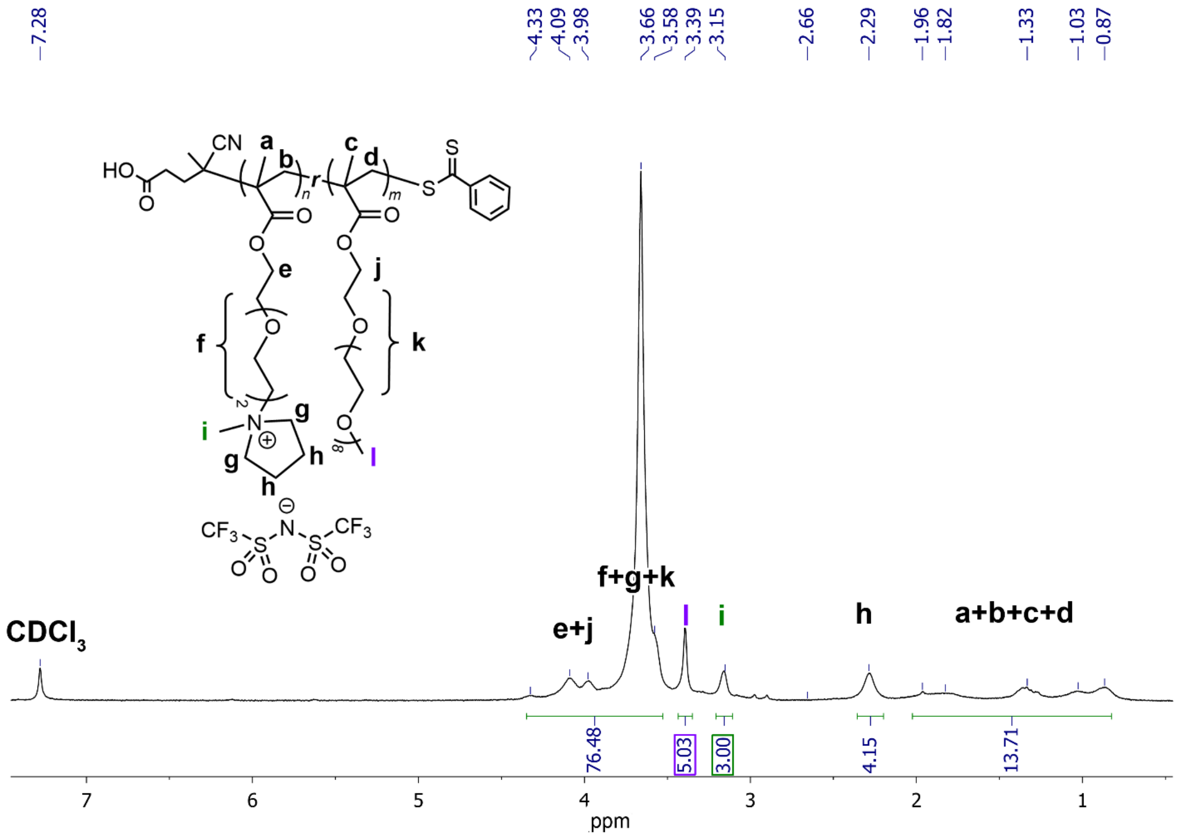

Figure S2. ^{1}H NMR of poly(ILMC$_{24}$-*r*-PEGM$_{40}$) in $CDCl_3$.

The proportion of ILMA (X_{ILMA}) and PEGM (X_{PEGM}) units in poly(ILMA-*r*-PEGM) was determined by ^{1}H NMR of isolated copolymer sample using the integrals of the signals at 3.39 ppm (-O-C<u>H</u>$_3$ from PEGM, see **j** on Figure S3) and 3.00 ppm (-C<u>H</u>$_2$-SO$_2$-N-SO$_2$CF$_3$ from ILMA, see **g** on Figure S3) and the equations S4 and S5:

$$X_{ILMA} = \frac{g}{g+j} \quad \text{(eq. S4)},$$

$$X_{PEGM} = \frac{j}{g+j} \quad \text{(eq. S5)},$$

PEGM : ILMA = X_{PEGM} / X_{ILMA}

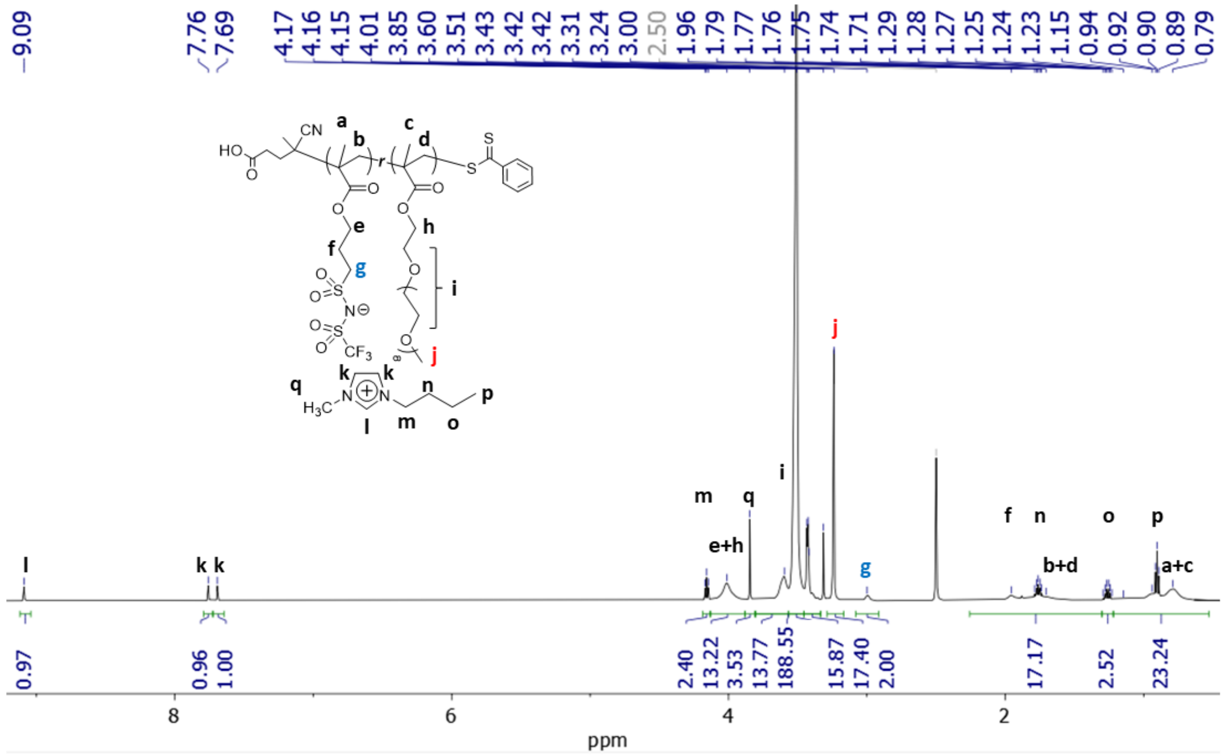

Figure S3. ^{1}H NMR of poly(ILMC$_{12}$-*r*-PEGM$_{68}$) in DMSO-d$_6$.

Determination of monomer units numbers and $M_{n(NMR)}$ in poly(ILM-r-PEGM) copolymers

The number of monomer units (**n** and **m**) and the $M_{n(NMR)}$ of the for poly(ILMC$_n$-*r*-PEGM$_m$) copolymer were defined by the equations S6-S8:

$\mathbf{n} = X_{ILMC} \times q \times \frac{[ILMC]_o+[PEGM]_o}{[CPAD]_o}$ (eq. S6),

$\mathbf{m} = X_{PEGM} \times q \times \frac{[ILMC]_o+[PEGM]_o}{[CPAD]_o}$ (eq. S7),

$M_{n(NMR)} = \mathbf{n} \times M_{ILMC} + \mathbf{m} \times M_{PEGM} + M_{CPAD}$ (eq. S8),

where X_{ILMC} and X_{PEGM} – the proportions of ILMC and PEGM units in the copolymer determined by equations S2 and S3; q – the total conversion of the monomers (Figure S1); $[ILMC]_o$, $[PEGM]_o$ and $[CPAD]_o$ are the initial concentrations of ILMC, PEGM monomers and chain transfer agent CPAD; M_{ILMC}, M_{PEGM} and M_{CPAD} – the molecular weights of monomers and CTA.

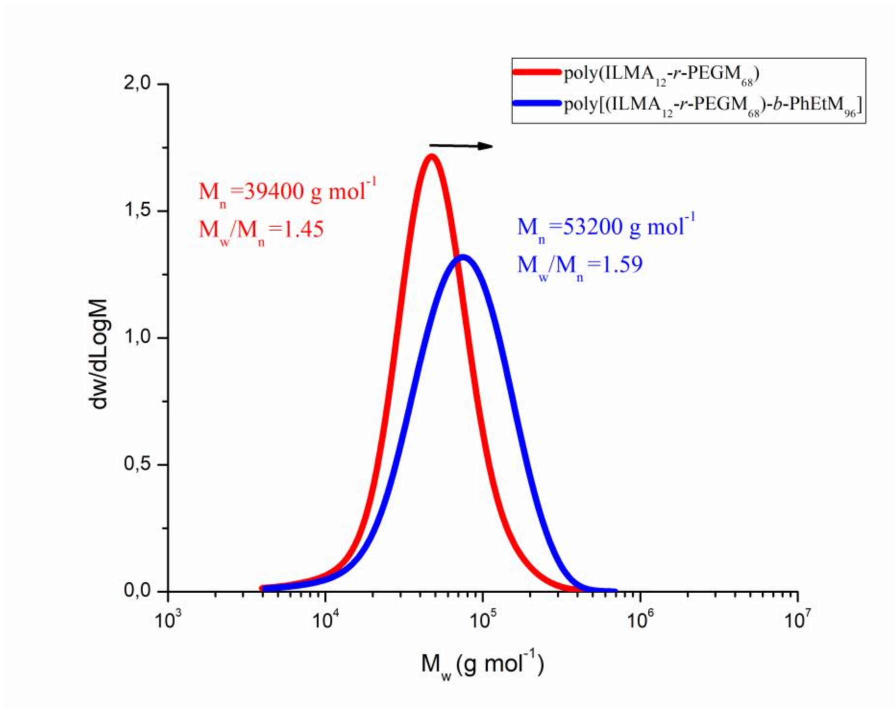

Figure S4. GPC traces of poly(ILMA$_{12}$-*r*-PEGM$_{68}$) and poly[(ILMA$_{12}$-*r*-PEGM$_{68}$)-*b*-PhEtM$_{96}$] copolymers (0.1 M LiTFSI in DMF, 50°C, PMMA standards).

Determination of monomers conversion (q) in the synthesis of poly[(ILM_n-r-$PEGM_m$)-b-$PhEtM_k$] block copolymers

For the calculation of total monomers conversion (q) the ^{1}H NMR spectra of the reaction mixture were recorded before and after polymerization. The solvent (DMF) was used as an internal standard. In ^{1}H NMR spectra of the reaction mixture before polymerization each of the integrals of $C=CH_2$ signals (a and a') from PhEtM monomer at 5.64 and 5.14 ppm were assigned as "1". At the same time the signal of DMF (H-CO-N$(CH_3)_2$, c) at 7.58 ppm was integrated. Then, in the ^{1}H NMR spectra of the reaction mixture after polymerization the signal of DMF (Figure S4, c) was integrated and set as the standard with the same value as before. The $C=CH_2$ signals of the residual monomer (Figure S4, b and b') were integrated and the conversion (q) was calculated in accordance with the equation (eq. S9):

$$q = \frac{2-(b+b')}{2} \quad \text{(eq. S9)}$$

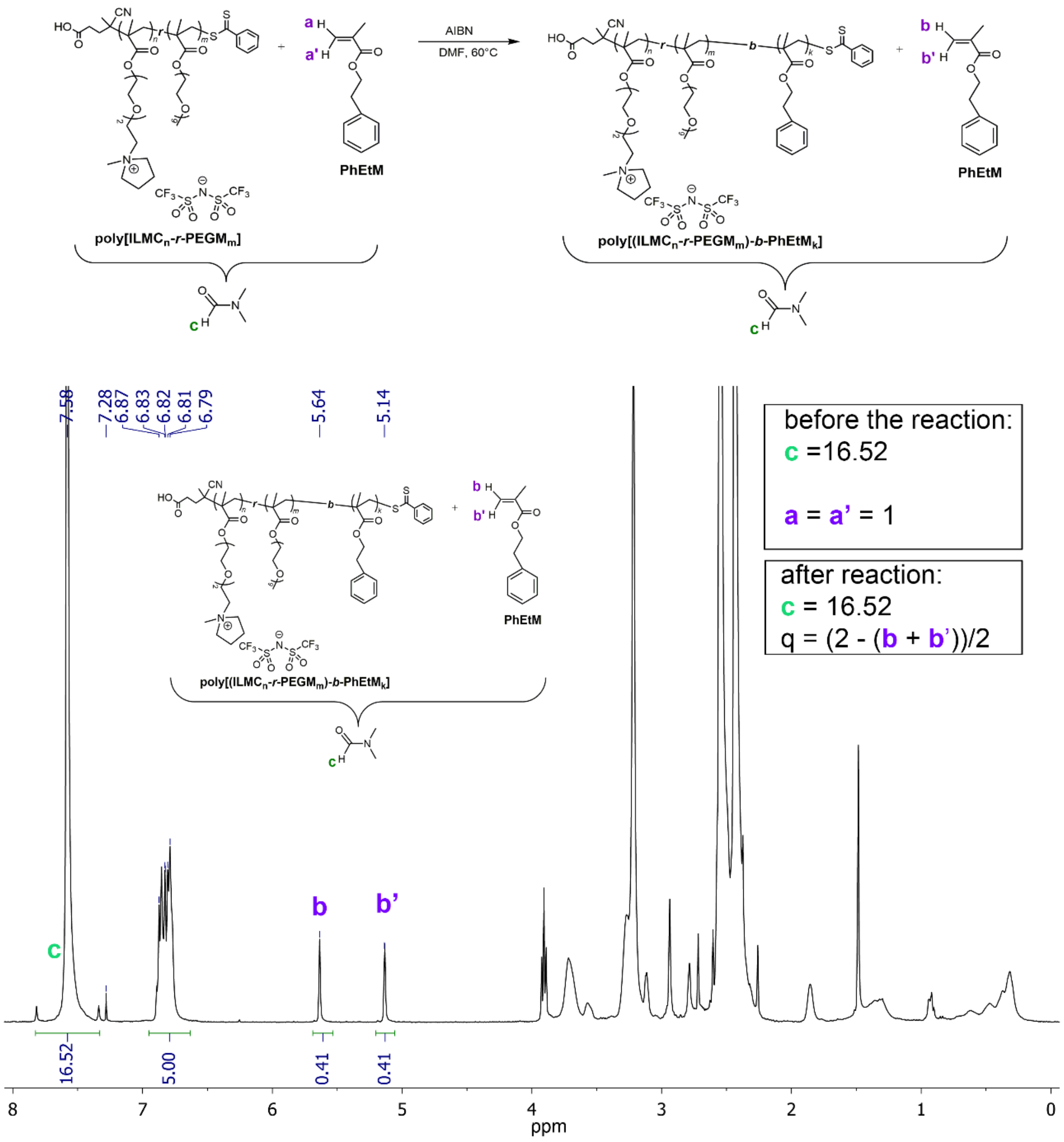

Figure S5. ^{1}H NMR of reaction mixture in $CDCl_3$ after the PhEtM copolymerization with poly(ILMC$_{24}$-*r*-PEGM$_{40}$) macro-CTA (poly[(ILM$_{24}$-r-PEGM$_{40}$)-b-PhEtM$_{95}$] synthesis).

Determination of PhEtM units number and $M_{n(NMR)}$ in poly[(ILM$_n$-r-PEGM$_m$)-b-PhEtM$_k$] block copolymers

The number of monomer units **k** in poly[(ILMC$_{24}$-*r*-PEGM$_{40}$)-*b*-PhEtM$_k$] was calculating using the following equation (eq. S10):

$$\boldsymbol{k} = n \times \frac{p/2}{k/3} \text{ (eq. S10)},$$

where k and n are the number of the PhEtM and ILMC units, respectively; p is the integral of -O-CH$_2$-C<u>H$_2$</u>-Ar signal from PhEtM monomer at 2.89 ppm (Figure S5, p); k is the integral of -N-C<u>H$_3$</u> signal from ILMC units at 3.14 ppm (Figure S5, k).

To check the correctness of the k determination, the following equation (eq. 11) can be used as well:

$$\boldsymbol{k} = q \times \frac{[PhEtM]_0}{[macro-CTA]_0} \text{ (eq. S11)},$$

where q is the PhEtM monomer conversion (defined by eq. S9), [PhEtM]$_o$ and [macro-CTA]$_o$ are the initial concentrations of PhEtM and poly(ILMC-*r*-PEGM) macro-CTA, respectively.

The $M_{n(NMR)}$ of poly[(ILM$_n$-*r*-PEGM$_m$)-*b*-PhEtM$_k$] block copolymers was defined by the simplified equation (eq. S12):

$$M_{n(NMR)} = \mathbf{k} \times M_{PhEtM} + M_{macro-CTA} \text{ (eq. S12)},$$

where q is the PhEtM monomer conversion (defined by eq. S9); M_{PhEtM} and $M_{macro-CTA}$ are the molecular weights of PhEtM monomer and poly(ILMC-*r*-PEGM) macro-CTA, respectively.

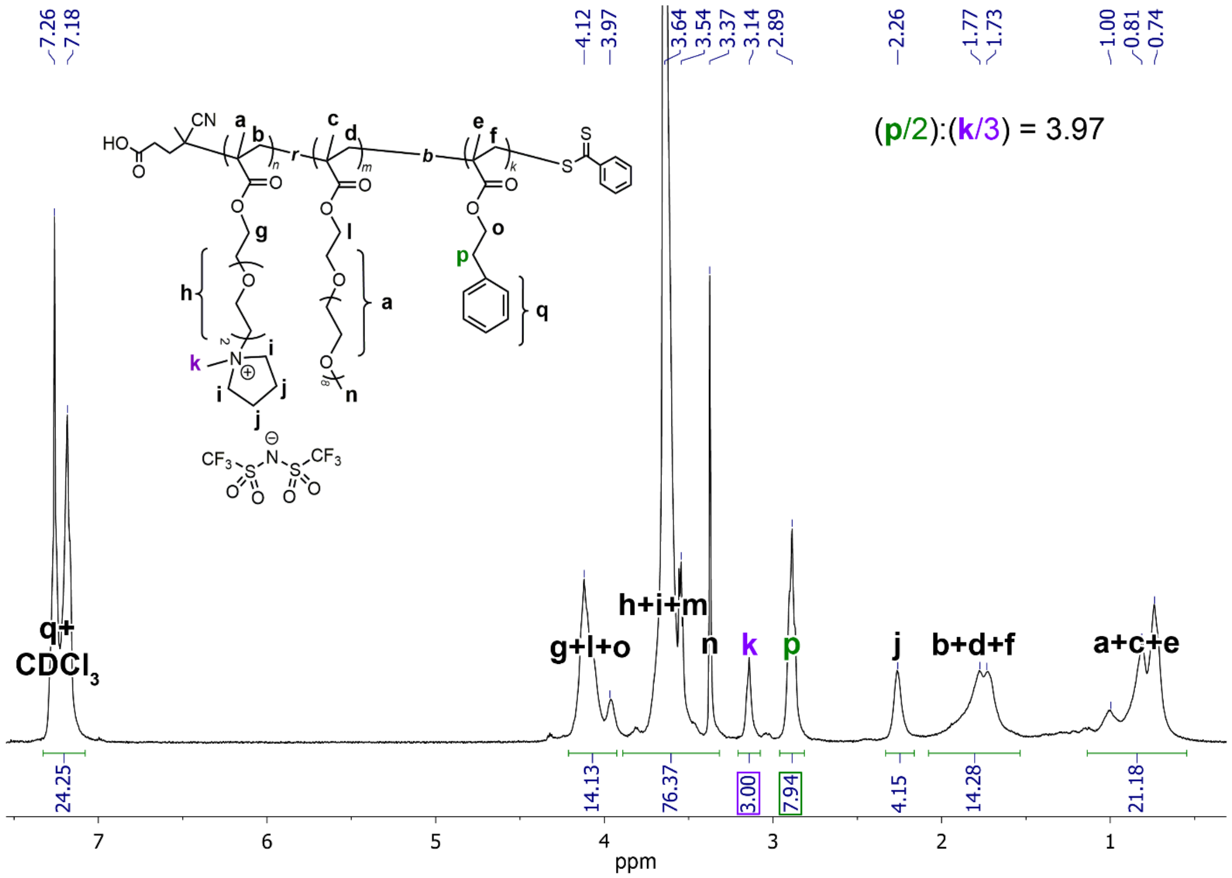

Figure S6. ^{1}H NMR of poly[(ILMC$_{24}$-*r*-PEGM$_{40}$)-*b*-PhEtM$_{95}$] in $CDCl_3$.

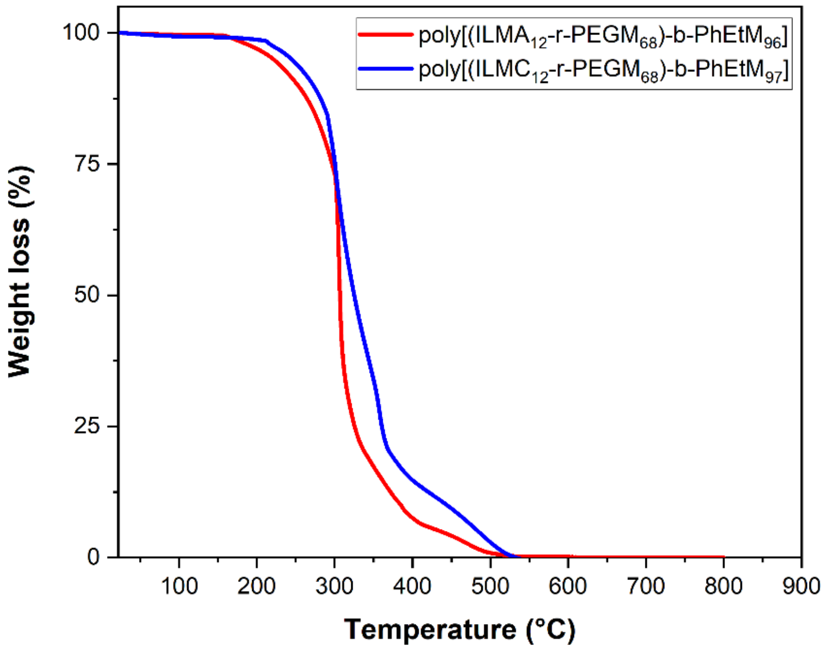

Figure S7. TGA traces of poly[(ILMA$_{12}$-*r*-PEGM$_{68}$)-*b*-PhEtM$_{96}$] and poly[(ILMC$_{12}$-*r*-PEGM$_{68}$)-*b*-PhEtM$_{97}$].

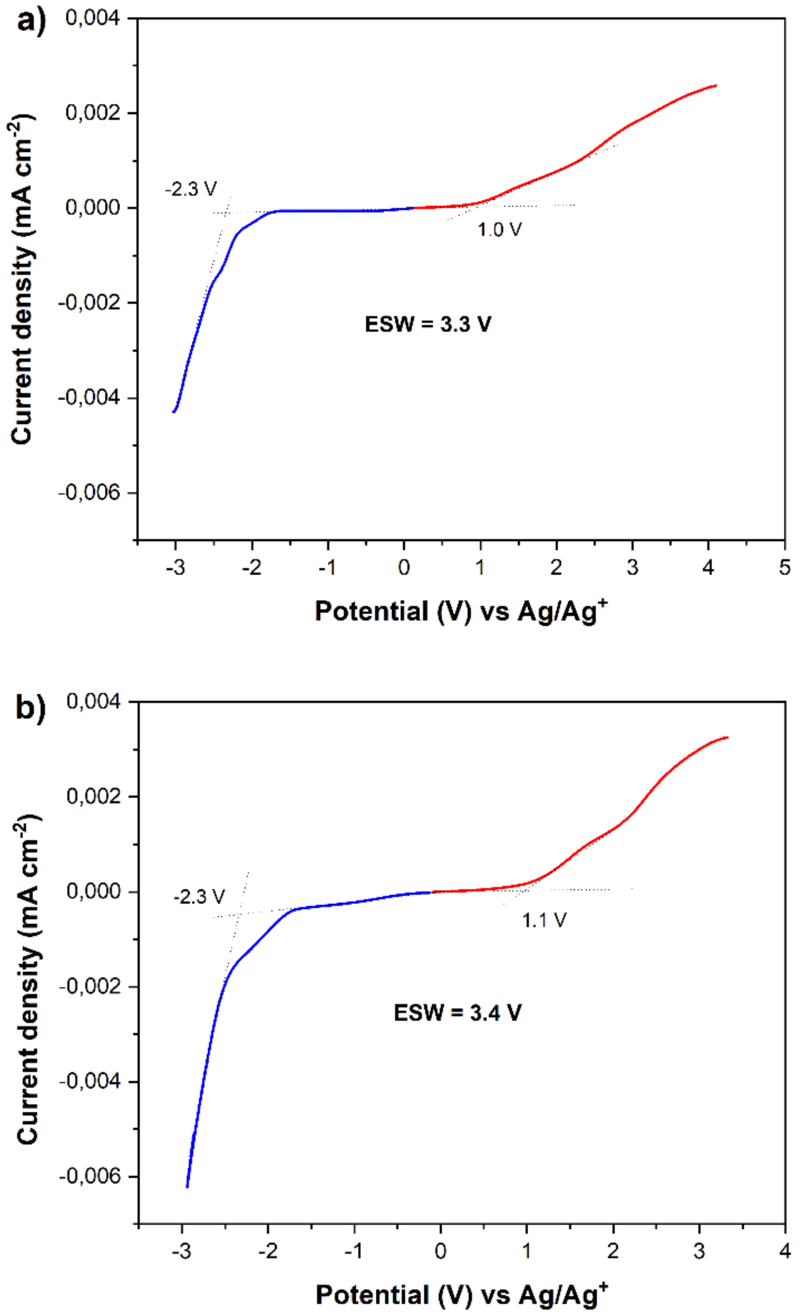

Figure S8. Electrochemical stability window for poly[(ILMC$_{12}$-*r*-PEGM$_{68}$)-*b*-PhEtM$_{97}$] (a) and poly[(ILMA$_{12}$-*r*-PEGM$_{68}$)-*b*-PhEtM$_{96}$] (b) obtained by CV at 25°C (Pt foils as working and counter electrodes, Ag mesh as pseudo-reference electrode, scan rate 5 mV/s).

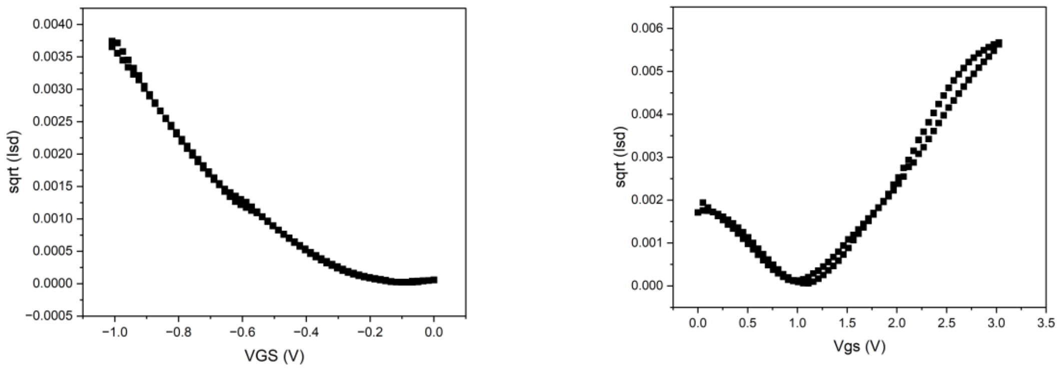

Figure S9. The squared root of source-drain current corresponding to the transfer curves reported in Fig 6 b,d.

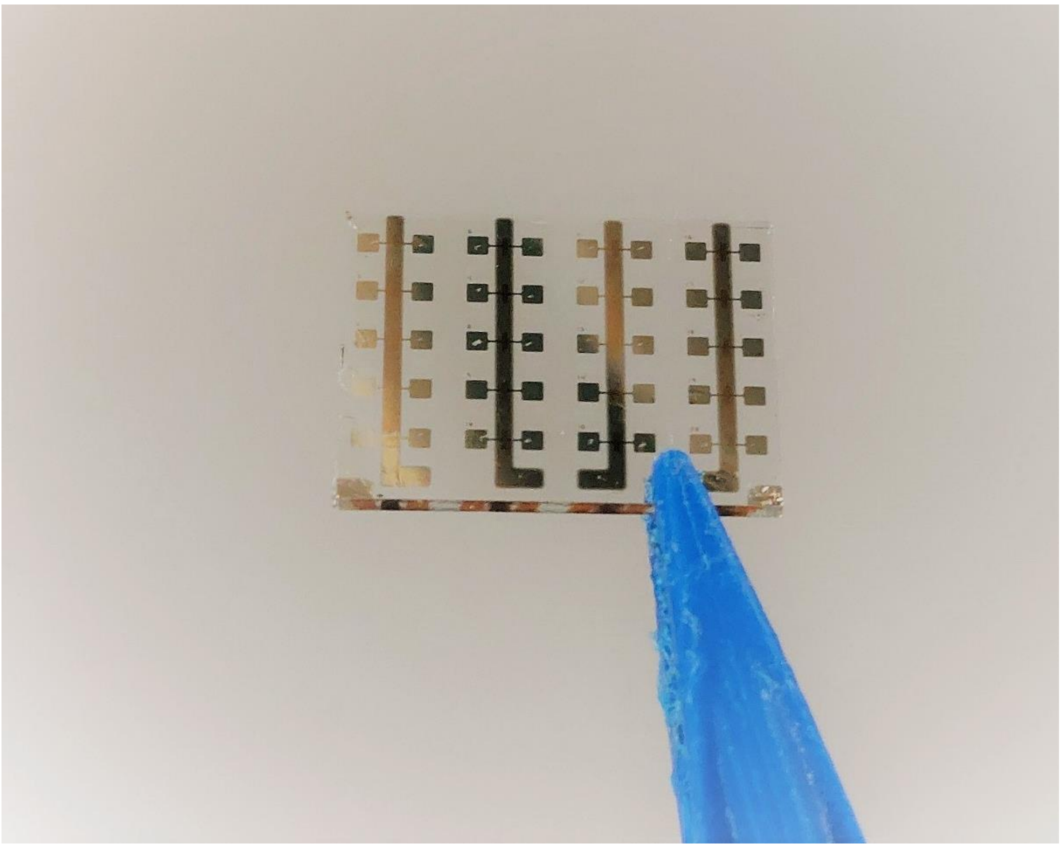

Figure S10. The image of the thin-film transistors fabricated.

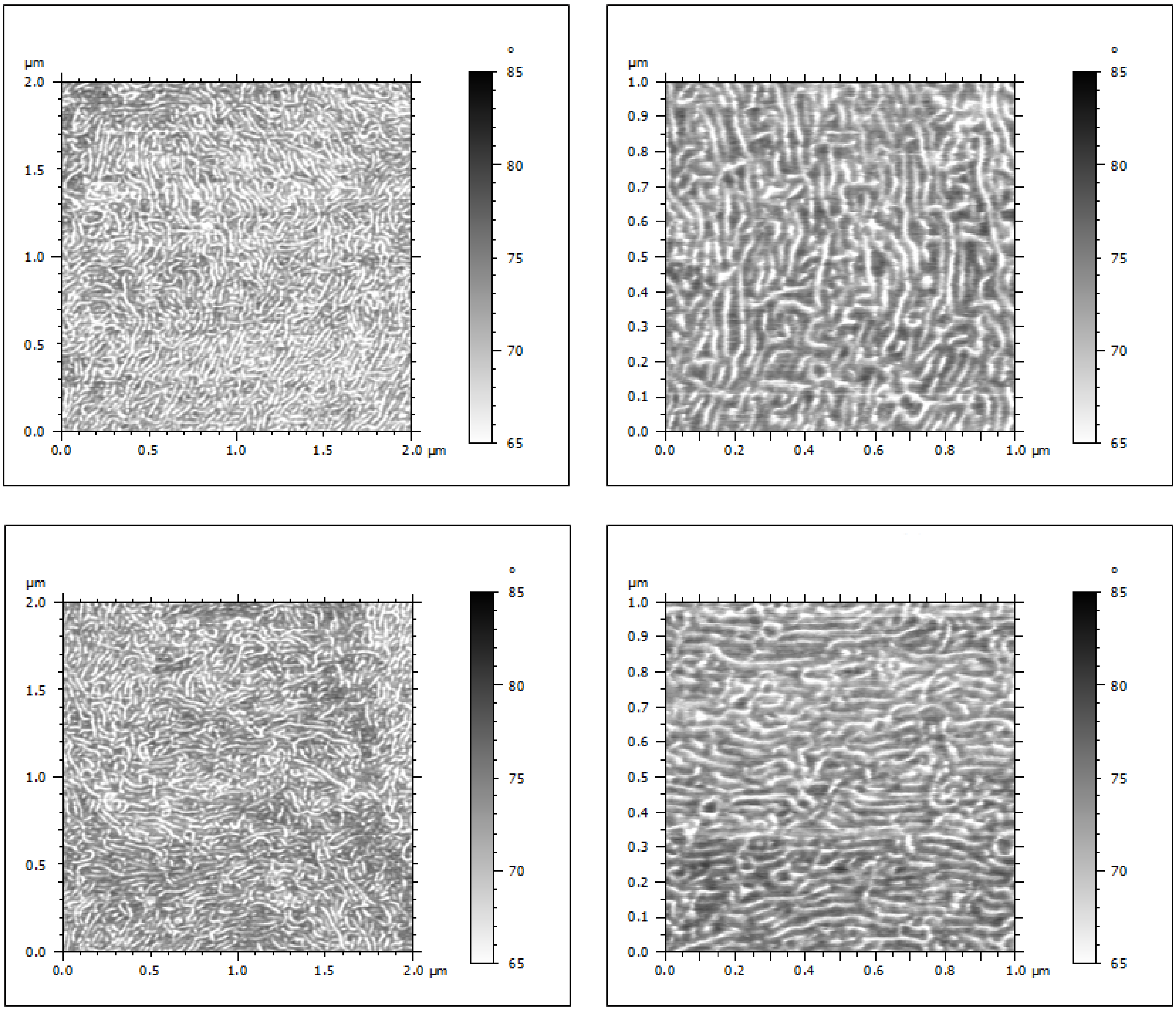

Figure S11. AFM images of **poly[(ILMA$_{12}$-*r*-PEGM$_{68}$)-*b*-PhEtM$_{96}$]** (**a**, **b**) films casted on golden foil.

References

(1) Puguan, J. M. C.; Boton, L. B.; Kim, H. Triazole-Based Ionene Exhibiting Tunable Structure and Ionic Conductivity Obtained via Cycloaddition Reaction: A New Polyelectrolyte for Electrochromic Devices. *Sol. Energy Mater. Sol. Cells* **2018**, *188*, 210–218. https://doi.org/10.1016/j.solmat.2018.09.009.

(2) Hu, H.; Yuan, W.; Jia, Z.; Baker, G. L. Ionic Liquid-Based Random Copolymers: A New Type of Polymer Electrolyte with Low Glass Transition Temperature. *RSC Adv.* **2015**, *5* (5), 3135–3140. https://doi.org/10.1039/C4RA13432J.

(3) Jourdain, A.; Serghei, A.; Drockenmuller, E. Enhanced Ionic Conductivity of a 1,2,3-Triazolium-Based Poly(Siloxane Ionic Liquid) Homopolymer. *ACS Macro Lett.* **2016**, *5* (11), 1283–1286. https://doi.org/10.1021/acsmacrolett.6b00761.

(4) Shaplov, A. S.; Lozinskaya, E. I.; Losada, R.; Wandrey, C.; Zdvizhkov, A. T.; Korlyukov, A. A.; Lyssenko, K. A.; Malyshkina, I. A.; Vygodskii, Y. S. Polymerization of the New Double-Charged Monomer Bis-1,3(N,N,N-Trimethylammonium Dicyanamide)-2-Propylmethacrylate and Ionic Conductivity of the Novel Polyelectrolytes. *Polym. Adv. Technol.* **2011**, *22* (4), 448–457. https://doi.org/10.1002/pat.1569.

(5) Wojnarowska, Z.; Feng, H.; Fu, Y.; Cheng, S.; Carroll, B.; Kumar, R.; Novikov, V. N.; Kisliuk, A. M.; Saito, T.; Kang, N.-G.; Mays, J. W.; Sokolov, A. P.; Bocharova, V. Effect of Chain Rigidity on the Decoupling of Ion Motion from Segmental Relaxation in Polymerized Ionic Liquids: Ambient and Elevated Pressure Studies. *Macromolecules* **2017**, *50* (17), 6710–6721. https://doi.org/10.1021/acs.macromol.7b01217.

(6) Ikeda, T. Poly(Ionic Liquid)s with Branched Side Chains: Polymer Design for Breaking the Conventional Record of Ionic Conductivity. *Polym. Chem.* **2021**, *12* (5), 711–718. https://doi.org/10.1039/D0PY01333A.

(7) Lee, M.; Choi, U. H.; Salas-de la Cruz, D.; Mittal, A.; Winey, K. I.; Colby, R. H.; Gibson, H. W. Imidazolium Polyesters: Structure–Property Relationships in Thermal Behavior, Ionic Conductivity, and Morphology. *Adv. Funct. Mater.* **2011**, *21* (4), 708–717. https://doi.org/10.1002/adfm.201001878.

(8) Lee, M.; Choi, U. H.; Colby, R. H.; Gibson, H. W. Ion Conduction in Imidazolium Acrylate Ionic Liquids and Their Polymers. *Chem. Mater.* **2010**, *22* (21), 5814–5822. https://doi.org/10.1021/cm101407d.

(9) Li, H.; Zhang, H.; Liao, X.; Sun, R.; Xie, M. Incorporating Trifunctional 1,6-Heptadiyne Moiety into Polyacetylene Ionomer for Improving Its Physical and Conductive Properties. *Polym. Chem.* **2020**, *11* (19), 3322–3331. https://doi.org/10.1039/D0PY00109K.

(10) Chen, M.; Dugger, J. W.; Li, X.; Wang, Y.; Kumar, R.; Meek, K. M.; Uhrig, D. W.; Browning, J. F.; Madsen, L. A.; Long, T. E.; Lokitz, B. S. Polymerized Ionic Liquids: Effects of Counter-Anions on Ion Conduction and Polymerization Kinetics. *J. Polym. Sci. Part A: Polym. Chem.* **2018**, *56* (13), 1346–1357. https://doi.org/10.1002/pola.29015.

(11) Cotessat, M.; Flachard, D.; Nosov, D.; Lozinskaya, E. I.; Ponkratov, D. O.; Schmidt, D. F.; Drockenmuller, E.; Shaplov, A. S. Effects of Repeat Unit Charge Density on the Physical and Electrochemical Properties of Novel Heterocationic Poly(Ionic Liquid)s. *New J. Chem.* **2021**, *45* (1), 53–65. https://doi.org/10.1039/D0NJ04143B.

(12) Drockenmuller, E.; Bernard, J.; Serghei, A.; Zhang, B.; Sood, R. Triethylene Glycol-Based Poly(1,2,3-Triazolium Acrylate)s with Enhanced Ionic Conductivity. *Polym. Chem.* **2015**, *6*, 3521–3528. https://doi.org/10.1039/C5PY00273G.

(13) Colliat-Dangus, G.; Obadia, M. M.; Vygodskii, Y. S.; Serghei, A.; Shaplov, A. S.; Drockenmuller, E. Unconventional Poly(Ionic Liquid)s Combining Motionless Main Chain 1,2,3-Triazolium Cations and High Ionic Conductivity. *Polym. Chem.* **2015**, *6* (23), 4299–4308. https://doi.org/10.1039/C5PY00526D.

(14) Shaplov, A. S.; Ponkratov, D. O.; Aubert, P.-H.; Lozinskaya, E. I.; Plesse, C.; Maziz, A.; Vlasov, P. S.; Vidal, F.; Vygodskii, Y. S. Truly Solid State Electrochromic Devices Constructed from Polymeric Ionic Liquids as Solid Electrolytes and Electrodes Formulated by Vapor Phase Polymerization of 3,4-Ethylenedioxythiophene. *Polymer* **2014**, *55* (16), 3385–3396. https://doi.org/10.1016/j.polymer.2014.04.013.

(15) Frenzel, F.; Guterman, R.; Anton, A. M.; Yuan, J.; Kremer, F. Molecular Dynamics and Charge Transport in Highly Conductive Polymeric Ionic Liquids. *Macromolecules* **2017**, *50* (10), 4022–4029. https://doi.org/10.1021/acs.macromol.7b00554.
